# Supplementary material for: Study protocol for a phase 1, randomised, double-blind, placebo-controlled study to investigate the safety, tolerability and pharmacokinetics of ascending topical doses of TCP-25 applied to epidermal suction blister wounds in healthy male and female volunteers
Source: BMJ Open. 2023 Feb 22;13(2):e064866. doi: 10.1136/bmjopen-2022-064866 (PMC9950920; doi:10.1136/bmjopen-2022-064866)
Supplement: Supplementary data [file bmjopen-2022-064866supp001.pdf]

Supplement 1, Full inclusion and exclusion criteria

Inclusion criteria	Exclusion criteria
1. Willing and able to give written informed consent for participation in the study.	1. History of any clinically significant disease or disorder which, in the opinion of the Investigator, may either put the subject at risk because of participation in the study, or influence the results or the subject's ability to participate in the study.
2. Healthy male or female subject 18-60 years (inclusive) of age at the time of signing the informed consent.	2. Disease that may interfere with wound healing, e.g., diabetes type I/II, arterial-, renal-, liver, or cardiac insufficiency, chronic obstructive lung disease, cancer, autoimmune disease, edema at the study site, severe obesity, or previous known wound healing problems, as judged by the investigator.
3. Body Mass Index (BMI) ≥ 18.0 and ≤ 30.0 kg/m2.	3. Active skin disease, e.g., dermatitis, psoriasis and wounds, and/or tattoos in the areas where suction blister wounds will be induced, as judged by the investigator.
4. Healthy and intact skin where the blister suction wounds will be induced.	4. Any planned major surgery within the duration of the study.
5. Women of childbearing potential (WOCBP) must have a documented negative serum pregnancy test done at the screening visit, within 4 weeks prior to suction blister formation and the start of study treatment.<br>WOCBP must practice abstinence (only allowed when this is the preferred and usual lifestyle of the subject) or must agree to use a highly effective method of contraception with a failure rate of < 1% to prevent pregnancy<br>Male subjects must be willing to use condom or be vasectomised or practice sexual abstinence to prevent pregnancy and drug exposure of a partner and refrain from donating sperm from the date of last dosing until 3 months after the last dosing with the IMP. Their female partner of child-bearing potential must use contraceptive methods with a failure rate of < 1% to prevent pregnancy (see above).	5. After 10 minutes supine rest at the time of screening, any vital signs values outside the following ranges:<br>• Systolic blood pressure 160 mmHg, or<br>• Diastolic blood pressure 100 mmHg, or<br>• Pulse 90 beats per minute (bpm)
6. Clinically relevant medical history, physical findings, vital signs, ECG and laboratory values at the time of screening, as judged by the Investigator.	6. Any clinically significant abnormalities in the resting ECG at the time of screening, as judged by the Investigator.
	7. Current smokers or users of nicotine products. Irregular use of nicotine (e.g., smoking, snuffing, chewing tobacco) less than three times per week is allowed before screening visit.
	8. Female subjects who are pregnant or lactating or planning a pregnancy.
	9. Systemic immunosuppressive treatment
	10. Subjects who are currently receiving or have received the following treatments within 2 weeks prior to screening are excluded from the

	study: - systemic corticosteroids or immunosuppressant agents; or - antibiotics via any route
	11. Regular use of anticoagulants (i.e., heparin, warfarin, coumarins, other anticoagulants per Investigator's judgement) or non-steroidal anti-inflammatory drugs (NSAIDs) within 2 weeks prior to the (first) administration of IMP, at the discretion of the Investigator.
	12. History of severe allergy/hypersensitivity or ongoing allergy/hypersensitivity, as judged by the Investigator, or history of hypersensitivity to drugs with a similar chemical structure or class to TCP-25 or to any excipients of the hydrogel.
	13. Planned treatment or treatment with another investigational drug within 3 months prior to Day -1.
	14. History of alcohol abuse or excessive intake of alcohol, as judged by the Investigator.
	15. Presence or history of drug abuse, as judged by the Investigator
	16. Plasma donation within one month of screening or blood donation (or corresponding blood loss) during the three months prior to screening.
	17. Involvement in the planning and/or conduct of the study.
	18. Investigator considers the subject unlikely to comply with study procedures, restrictions and requirements.

Supplement 2, Restrictions during the study

- Contraception Requirements: The male volunteers are expected to use condom to prevent pregnancy and drug exposure of a partners and refrain from donating sperm from the date of dosing until 4 weeks after the last dosing of the IMP.
  Fertile female study subjects are expected to practice abstinence or use contraceptive methods with a failure rate of < 1% to prevent pregnancy during the same period, i.e., combined [oestrogen and progestogen containing] hormonal contraception associated with inhibition of ovulation [oral, intravaginal, transdermal], progestogen-only hormonal contraception associated with inhibition of ovulation [oral, injectable, implantable], IUD or intrauterine IUS) from at least 4 weeks prior to dose to 4 weeks after last dose.
- Caffeine: Consumption of caffeine equivalent to up to 5 cups of coffee per day will be allowed from screening to the end-of-study visit.
- Other xanthine or taurine containing products/beverages than tea or coffee: Energy drinks (e.g., Red Bull, Battery, Flying Horse, La Bomba) are not allowed from screening to the end-of-study visit.
- Nicotine: Smoking (>3 cigarettes per day) or use of nicotine-containing products is not allowed from screening to the end-of-study visit.
- Exercise: From Day 1 to Day 11, the subjects must abstain from strenuous exercise that would cause excessive sweating compromising the dressing of the wounds.
- Blood donation: The subjects must not donate blood or plasma during the study until 3 months after the final medical examination at the end-of-study visit.
- Participation in other clinical studies: Study subjects are not allowed to participate in any other interventional clinical study during the study period.

Supplement 3, Prior and concomitant therapy

Prohibited medication

From 2 weeks prior to wounding and throughout the study until Visit 9 (unless indicated for treatment of any AEs):

- Systemic corticosteroids or immunosuppressant agents; or
- Antibiotics via any route
- Anticoagulants (e.g., heparin, warfarin, coumarins, other anticoagulants per Investigator’s judgement)
- Regular use of NSAIDs (i.e., ibuprofen, diclofenac, acetylsalicylic acid and others)

Allowed medication

- Paracetamol 1 g up to 4 times daily is allowed for treatment of post-procedural pain.
- Any medications at a stable dose to treat medical history events will be allowed throughout the study.
- Medications considered necessary for the subject´s safety and wellbeing may be given at the discretion of the Investigator during the study. Following consultation with the Sponsor, the Investigator will determine whether or not the subject should continue in the study.

Supplement 4, General withdrawal criteria

Subjects are free to discontinue their participation in the study at any time and for whatever reason without affecting their right to an appropriate follow-up investigation or their future care. If possible, the reason for withdrawal of consent should be documented.

Subjects may be discontinued from the study at any time at the discretion of the Investigator.

Reasons for discontinuation include:

- Subject decision
- Severe non-compliance to study protocol procedures, as judged by the Investigator and/or Sponsor
- Subject is lost to follow-up.
- Significant AEs posing a risk for the subject, as judged by the Investigator and/or Sponsor
- Withdrawal of informed consent to the use of biological samples
- Pregnancy
- Death
- Meeting of an exclusion criterion during the study, which, in the opinion of the Investigator, may pose a risk for the subject
- Use of prohibited medication

Supplement 5 – text for recruitment and advertisement approved by the ethics committee

Long version:

En fas 1, randomiserad, dubbelblind och kontrollerad studie på friska män och kvinnor för att undersöka säkerhet, tolerans och exponering efter applikation av stigande doser av TCP-25, en gel som appliceras utvärtes på ytliga sår i huden

Syftet med denna Fas 1 studie är att undersöka läkemedlets säkerhet och tolerans

Kriterier för deltagande:

• Du ska vara frisk

• Du får ej ha högt blodtryck eller diabetes

• Du får inte ha födelsemärken, tatueringar eller hudförändringar på låren.

• Du får inte vara överviktig eller underviktig (BMI ska ligga mellan 18–30 kg/m2)

• Du får ej röka/snusa regelbundet

• Du får ej ha deltagit i annan läkemedelsstudie senaste 3 månaderna

• Du får inte ha lämnat plasma 1 månad eller blod 3 månader innan den inledande hälsokontrollen för studien.

• Om du är i fertil ålder och inte steril måste du 4 veckor efter dosering av studieläkemedel använda dubbelbarriär vid heterosexuella samlag, dvs kondom/godkända preventivmedel. Är du kvinna gäller detta även fyra veckor innan studiestart.

I studien sker totalt 8 besök på Klinisk Prövningsenhet, Skånes Universitetssjukhus i Lund, under en period av 15 dagar. Du kommer få 2 små blåsor, 2 på vardera lår (totalt 4 blåsor).

Blåsorna skapas med hjälp av en pump som skapar undertryck och liknas de blåsor som uppstår vid skoskav både till utseende och djup. Blåstaket och vätskan avlägsnas och ett sår med en diameter på 1 cm uppstår, studieläkemedel/placebo ges sedan lokalt på såren.

Du får ersättning för ditt deltagande. Vill du delta eller ha mer information? Maila provningsenheten.forskningsstudie@skane.se eller besök https://sodrasjukvardsregionen.se/kliniskastudier/delta-i-klinisk-studie/ Studien är godkänd av Etikprövningsmyndigheten och Läkemedelsverket. Din medverkan är frivillig och du kan när som helst avbryta ditt deltagande. Den information du lämnar kommer att behandlas i enlighet med Dataskyddsförordningen (GDPR).

Short version:

En fas 1, randomiserad, dubbelblind och kontrollerad studie på friska män och kvinnor för att undersöka säkerhet, tolerans och exponering efter applikation av stigande doser av TCP-25, en gel som appliceras utvärtes på ytliga sår i huden

Syftet med studien är att undersöka läkemedlets säkerhet och tolerans samt hur läkemedlet tas upp, fördelas och utsöndras i kroppen.

Du får ersättning för ditt deltagande samt reseersättning.

För att kunna delta behöver du vara frisk och ej ha några allvarliga sjukdomar. Vill du delta eller ha mer information? Maila provningsenheten.forskningsstudie@skane.se eller besök https://sodrasjukvardsregionen.se/kliniskastudier/delta-i-klinisk-studie/

Studien är godkänd av Etikprövningsmyndigheten och Läkemedelsverket. Din medverkan är frivillig och du kan när som helst avbryta ditt deltagande. Den information du lämnar kommer att behandlas i enlighet med Dataskyddsförordningen (GDPR).
