# Supplementary material for: Study protocol for a phase 1, randomised, double-blind, placebo-controlled study to investigate the safety, tolerability and pharmacokinetics of ascending topical doses of TCP-25 applied to epidermal suction blister wounds in healthy male and female volunteers
Source: BMJ Open. 2023 Feb 22;13(2):e064866. doi: 10.1136/bmjopen-2022-064866 (PMC9950920; doi:10.1136/bmjopen-2022-064866)
Supplement: Author's manuscript [file bmjopen-2022-064866.draft_revisions.pdf]

BMJ Open is committed to open peer review. As part of this commitment we make the peer review history of every article we publish publicly available.

When an article is published we post the peer reviewers' comments and the authors' responses online. We also post the versions of the paper that were used during peer review. These are the versions that the peer review comments apply to.

The versions of the paper that follow are the versions that were submitted during the peer review process. They are not the versions of record or the final published versions. They should not be cited or distributed as the published version of this manuscript.

BMJ Open is an open access journal and the full, final, typeset and author-corrected version of record of the manuscript is available on our site with no access controls, subscription charges or pay-per-view fees (http://bmjopen.bmj.com).

If you have any questions on BMJ Open's open peer review process please email
info.bmjopen@bmj.com

BMJ Open

Study protocol for a Phase 1, Randomized, Double-Blind, Placebo-Controlled Study to Investigate the Safety, Tolerability, and Pharmacokinetics of Ascending Topical Doses of TCP-25 Applied to Epidermal Suction Blister Wounds in Healthy Male and Female Volunteers

Journal:	*BMJ Open*
Manuscript ID	bmjopen-2022-064866
Article Type:	Protocol
Date Submitted by the Author:	16-May-2022

Complete List of Authors:	Lundgren, Sigrid; Lund University, Department of Clinical Sciences Lund; Skåne University Hospital Lund, Department of Dermatology<br>Wallblom, Karl; Lund University, Department of Clinical Sciences Lund; Skåne University Hospital Lund, Department of Dermatology<br>Fisher, Jane; Lund University, Department of Clinical Sciences Lund<br>Erdmann, Susanne; Skåne University Hospital Lund, Department of Dermatology<br>Smidtchen, Artur; Lund University, Department of Clinical Sciences Lund; Skåne University Hospital Lund, Department of Dermatology<br>Saleh, Karim; Lund University, Department of Clinical Sciences Lund; Skåne University Hospital Lund, Department of Dermatology

Keywords:	WOUND MANAGEMENT, Clinical trials < THERAPEUTICS, DERMATOLOGY

SCHOLARONE™
Manuscripts

Study protocol for a Phase 1, Randomized, Double-Blind, Placebo-Controlled Study to Investigate the Safety, Tolerability, and Pharmacokinetics of Ascending Topical Doses of TCP-25 Applied to Epidermal Suction Blister Wounds in Healthy Male and Female Volunteers

Sigrid Lundgren[1, 2*a], Karl Wallblom[1, 2*], Jane Fisher[1], Susanne Erdmann[2], Artur Schmidtchen[1, 2, 3], Karim Saleh[1, 2].

Affiliations:

[1] Section for Dermatology and Venereology, Department of Clinical Sciences Lund, Lund University, Lund, Sweden

[2] Department of Dermatology, Skane University Hospital, Lund, Sweden

[3] Copenhagen Wound Healing Center, Bispebjerg Hospital, Department of Biomedical Sciences, University of Copenhagen, Copenhagen, Denmark

* Equal contribution

[a] Corresponding author

Email addresses:

Sigrid Lundgren: sigrid.lundgren@med.lu.se

Karl Wallblom: karl.wallblom@gmail.com

Jane Fisher: jane.fisher@med.lu.se

Susanne Erdmann: susanne.erdman@banhof.se

Artur Schmidtchen: artur.schmidtchen@med.lu.se>

Karim Saleh: karim.saleh@med.lu.se

Word Count: 3612 words

Key words: Wounds and injuries; Wound infection; Wound Healing

For peer review only

Abstract

Introduction TCP-25 gel is intended to be used for treatment of infection and inflammation in wounds. Current local therapies for wounds have limited efficacy to prevent infections and there are no wound treatments available today that target the excessive inflammation that often hampers wound healing in both acute and chronic wounds. There is therefore a high medical need for new therapeutic alternatives.

Methods and analysis

A randomised, double-blinded, first-in-human study was designed to evaluate the safety, tolerability, and potential systemic exposure of three increasing doses of the TCP-25 gel applied topically on suction blister wounds in healthy adults. The dose-escalation will be divided in 3 sequential groups with 8 subjects in each group (24 patients in total). Within each cohort, the subjects will receive TCP-25 on one wound on each thigh and placebo on one wound on each thigh in a randomised fashion, with a reverse localisation on the respective thigh of control and treatment over 8 days (total of 5 doses). An internal safety review committee will monitor emerging safety and plasma concentration data over the course of the study and must give a favourable recommendation prior to initiating dose groups 2 and 3.

Ethics and dissemination The study will be performed in accordance with ethical principles consistent with the Declaration of Helsinki, ICH/GCPE6 (R2), EU Clinical Trials Directive, and applicable local regulatory requirements.

This study is approved by the Swedish Medical Products Agency and the Swedish ethics committee under the registration number 2022-00527-01. The results of this study will be disseminated via publication to a peer-reviewed journal at the discretion of the sponsor.

Trial registration number: EudraCT 2021-004728-14, Clinical trials.gov (pending release)

Strengths and limitations of this study

- This is a first-in-human study of a drug that simultaneously targets bacteria and inflammation
- The choice of the suction blister technique is a strength because it is minimally invasive and provides a consistent, though limited, wound area
- The study includes rigorous testing and monitoring and features an internal safety review committee which evaluates the data before each dose increase, ensuring safety

For peer review only - http://bmjopen.bmj.com/site/about/guidelines.xhtml

- Frequent outcomes assessment is a strength, but could limit the study because it is time-consuming and burdensome for participants
- Limitations include the fact that this is a single-centre study with a limited number of participants, and includes only a specific age range, thus limiting its applicability to other populations.

Introduction

In normal conditions, wounds heal in a sequenced and timely manner, characterised by four major phases (haemostasis, inflammation, proliferation, and remodelling). This is a complex process involving chemokines, growth factors, cytokines, proteases and antiproteases, and multiple cell types that all work in a controlled manner during the healing process. This timely process can be interrupted at various stages and by different factors, leading to a dysfunctional inflammatory phase and disrupting wound healing. This common feature characterises most major wound complications. (1)

Wounds of various types have a significant impact on patients, health care, and society (1). Types of wounds include acute post-surgical wounds and burns, and non-healing wounds resulting from diabetes or circulatory disturbances (2). It is critical both to prevent and treat infections and to address excessive inflammation in various wound types. To our knowledge there is currently no available wound treatment that targets both the infection and the excessive inflammation simultaneously. Furthermore, as antimicrobial resistance increases, common antibiotics and some antiseptics are becoming even less effective in treating wound infections. Antimicrobial resistant bacteria are often present in various wounds, thus increasing the risk of antimicrobial resistant infections (3-5). Therefore, there is a great need for new and cost-effective treatments that will improve healing and reduce both infection and inflammation in patients with various wounds.

TCP-25 is a new class of peptide-based drug which has a dual action targeting both infection and the associated excessive inflammation. A hydrogel containing TCP-25 treated infection and inflammation in murine and porcine wound infection models leading, hastening healing of infected wounds (6, 7). The mode of action is well characterised and involves interactions with bacterial lipopolysaccharide (LPS), other bacterial products, and the cell-receptor CD14, enabling a down-modulation of inflammation, while simultaneously exerting direct

For peer review only

antimicrobial effects. Importantly, TCP-25 acts on multiple multi-drug resistant bacterial isolates (6).

Suction blister wounds are standardised with respect to wound depth, as the entire epidermal layer is ablated and the dermis is exposed, size, and closure time (8). The mechanical nonbleeding excoriation elicits an innate immune response accompanied by increased blood flow and inflammatory cell infiltration in the underlying dermis. Epidermal regeneration occurs from the edges and appendages (9). Usually, the wounds are colonised by commensal bacteria after some days (10).

The safety and tolerability of three dose levels of TCP-25 gel will be tested to monitor safety in experimental wounds generated by the suction blister technique. It will be compared to a placebo control gel which is identical to TCP-25 gel, without the active ingredient (TCP-25). An overview of trial registration data is summarized in **Table 1**.

Study objectives

The primary objective of this study is to evaluate the safety and tolerability of three dose levels of TCP-25 gel (0.86 mg/mL, 2.9 mg/mL and 8.6 mg/mL) applied topically to epidermal suction blister wounds. **The secondary objective** is to evaluate the systemic exposure of TCP-25 after topical application to epidermal suction blister wounds.

Exploratory objectives include evaluation of wounds from photographs, and to collect and store samples from dressings and wound surfaces for future exploratory research. The exploratory analyses will be described in a separate report.

Methods and analysis:

Trial design and setting

This trial is a randomised, double-blinded, FIH study designed to evaluate the safety, tolerability, and potential systemic exposure of three increasing doses of the TCP-25 gel applied topically on suction blister wounds in healthy adults. The study will run as a single-centre study, conducted at Skåne University Hospital in Lund, Sweden. Screening and enrolment started on April 7, 2022. Randomization and treatment administration started on April 25, 2022. The final follow-up for the last participant in the initial target sample size (24 participants) is estimated to be at the end of June 2022, with the possibility of extension beyond this date to include more participants. The dose-escalation will be divided into 3 sequential groups with 8 subjects in each group. Within each dose group, each subject will

For peer review only - http://bmjopen.bmj.com/site/about/guidelines.xhtml

For peer review only

receive TCP-25 on one wound on each thigh and placebo on one wound on each thigh in a randomised manner, but with a reverse reciprocal position on each respective thigh, to a total of 5 doses over 8 days. An internal safety review committee (iSRC) will monitor emerging safety and plasma concentration data over the course of the study and must give a favourable recommendation prior to initiating the higher dose in groups 2 and 3, based on safety parameters as described below. **Figure 1** presents an overview of the study design and which interventions and testing will be done on each day.

Eligibility criteria

Prior to enrolment in the study, all subjects will be screened. Subjects will only be included if they fulfil all of the inclusion criteria and none of the exclusion criteria. A summary of the eligibility criteria is listed in **Table 2**. Details of the eligibility criteria, prior and concomitant therapy restrictions, and general restrictions during the study can be found in **Supplements 1,2, and 3.**

Consent and withdrawal

All subjects will be provided with written and oral information about the trial, including collection of biological specimens for exploratory studies, by the investigator or authorized associate, and given sufficient time to make an informed decision about participation. Informed consent must be signed and documented before any study-specific procedures are performed.

Subjects will be free to discontinue their participation in the study at any time and for whatever reason without affecting their right to an appropriate follow-up investigation or their future care. If possible, the reason for withdrawal of consent shall be documented. Subjects may also be discontinued from the study at any time at the discretion of the Investigator according to the criteria described in **Supplement 4**. A subject who prematurely discontinues participation in the study will be asked about the presence of any adverse events and the Investigator must ask the subject if he/she is willing to be assessed according to the procedures scheduled for the end-of-study visit. Any ongoing adverse events will be followed until they are resolved.

Suction blister wound formation

Blister wounds will be formed on the medial aspect of the thigh, two blisters on each leg with a 6 cm distance in between them. Before wounding, hair at the wound sites will be shaved.

For peer review only - http://bmjopen.bmj.com/site/about/guidelines.xhtml

Each wound site will be wiped with an ethanol-soaked gauze. The exact wound sites will be marked using a skin marker.

The blisters will be made using the Model NP-4 (Electronic Diversities, Finksburg, MD) suctioning device (11) operating in the negative pressure range 8-16 inchHg with heating for optimal blister formation. Blister wound diameter will be 10 mm. The device will run until all blisters have formed, which takes approximately 60-70 minutes. The roof of the blister will be excised using a sterile forceps and scissors.

Interventions

Treatment: Each wound will receive 0.15 mL of a sterile gel containing either TCP-25 (0.86, 2.9, or 8.6 mg/mL according to the dose group) or placebo (0 mg/mL TCP-25), applied topically.

Dressing: After application of the assigned intervention, each wound will be dressed using a 2 x 2 cm Mepilex transfer (Mölnlycke health care) as primary dressing and covered with a secondary dressing (Tegaderm, 3M™). A secondary protective layer will be applied, consisting of overlapping gauze swabs covered with a secondary Tegaderm dressing.

Outcomes

The follow-up period will be for 11 days after application of the intervention. This short time period ensures participant retention and complete follow-up.

The primary objective of safety and tolerability will be evaluated using the following **primary endpoints:**

- Frequency, intensity, and seriousness of adverse events (AEs).
- Local tolerability by evaluating the incidence of abnormal local reactions as compared to expected wound healing outcome.
- Clinically significant changes from baseline in electrocardiogram (ECG), vital signs (systolic/diastolic blood pressure, pulse rate), safety laboratory parameters (haematology, clinical chemistry, and coagulation, described in detail in **Table 3**) and physical examinations.

The secondary objective of systemic exposure will be evaluated using the following **secondary endpoint:**

- TCP-25 plasma concentrations as measured at Day 1, Day 2, Day 3 and Day 5.

For peer review only - http://bmjopen.bmj.com/site/about/guidelines.xhtml

Data collection

Local tolerability: The investigator will evaluate tolerability by noting abnormal reactions by direct assessment of:

- Skin and wound erythema (abnormal reactions noted)
- Skin and wound oedema and swelling (abnormal reactions noted)
- Wound necrosis, crusting, and haemorrhage (abnormal reactions noted)
- Wound purulent discharge as sign of excessive bacterial colonization and/or infection

The result (yes/no) for each parameter and time point will be documented in the eCRF. Local tolerability parameters, which are judged by the investigator to be aggravated compared to what is expected for blister-induced epidermal wounds, will be reported as AEs.

ECG: Single 12-lead ECG will be recorded in supine position after 10 minutes of rest using an ECG machine. Heart rate (HR), PR, QRS, and QT intervals, and corrected QT interval by Fredericia (QTcF) will be recorded. Safety ECGs will be reviewed and interpreted on-site by the investigator. Any abnormalities will be specified and documented as not clinically significant or clinically significant. Abnormal post-dose findings assessed by the investigator as clinically significant will be reported as AEs.

Vital signs: Will be assessed in the sitting position after 10 minutes of rest. Any vital signs outside the normal ranges will be judged as not clinically significant or clinically significant. The assessment will be recorded in the eCRF. Vital signs judged by the investigator as “abnormal, clinically significant” after application of the intervention will be reported as AEs.

Physical examination: A physical examination will include assessments of general condition, lymph nodes, throat, heart, lungs, and abdomen. Any abnormalities will be specified and documented as clinically significant or not clinically significant. Abnormal post-dose findings assessed by the Investigator as clinically significant will be reported as AEs.

Safety lab measurements: Blood will be collected with a peripheral venous catheter using the standard clinical operating procedure and measured by the clinical chemistry department at Skåne University Hospital, Lund, using standard clinical assays. The safety laboratory parameters measured are detailed in **table 1**.

For peer review only - http://bmjopen.bmj.com/site/about/guidelines.xhtml

Adverse events: The grading of the intensity of AEs will follow the Common Terminology Criteria for Adverse Events v5.0 [25]. All AEs, including those AEs defined above, shall be recorded. AE will be assessed for causality and severity by the investigator, and any suspected unexpected severe adverse reactions will be reported to the local competent authority and ethics committee according to local and European regulations.

These interventions and measurements will be done according to the schedule presented in **Figure 1.**

Sample size and recruitment

No formal sample size calculation has been performed for this study because the study objectives involve safety with few events expected. The proposed sample size is considered sufficient to provide adequate information for the study objectives. Approximately 50 subjects will be screened to achieve 24 randomised and evaluable subjects. The subjects will be healthy volunteers recruited from the clinic's database of healthy volunteers and by advertisement. The advertisement texts approved by the independent ethics committee (IEC) will be used to create all the materials (digital, radio and/or print) for recruitment.

Allocation

Each subject will have four suction blister wounds, two on each thigh. Wounds will be labelled R1 (right thigh, distal), R2 (right thigh, proximal), L1 (left thigh, distal) and L2 (left thigh, proximal).

A computer-generated randomization list will be created using SAS Proc Plan, SAS Version 9.4. The randomization list will contain subject number, wound position, thigh (left/right) and treatment and will be kept by the randomizer in a sealed envelope until database lock. On Day 1 the subjects in each dose group (n=8) will be randomised with regards to the treatment allocation of each wound. Two wounds per patient will receive TCP-25, and the other two will receive placebo. The proximal wound (R1 or L1) of one thigh and the distal wound of the other thigh (L2 or R2) will receive the same treatment, according to the assigned treatment allocation. Two syringes per patient will be prepared by an unblinded nurse in a nearby medical room, one with TCP-25 and one with placebo, labelled with the randomisation number of the patient and the identification of two wounds (R1 + L2 or R2 +

For peer review only

L1) according to the assigned treatment allocation. These pre-filled and labelled syringes will be provided to the blinded research staff for application to the assigned wounds.

If needed for emergency unblinding, sealed individual treatment code envelopes will be kept at the clinic and at a contract research organisation (Clinical Trial Consultants AB's Pharmacovigilance department) in locked and restricted areas.

Blinding

This is a double-blind study and the allocation of treatments will not be disclosed until clean file has been declared and the database has been locked. The TCP-25 and placebo gels are identical in appearance.

Unblinding will only happen in the following situations:

1. The treatment code may only be broken by the Principal Investigator or delegate in case of emergency when knowledge of the treatment received is necessary for the proper medical management of the subject.
2. The randomization code may be broken by the iSRC during the assessment process (partial unblinding) to enable their decision on continued dosing of further cohorts. The medical staff and subjects will remain blinded to the treatment allocation.

Data management

The data management routines include procedures for handling of the eCRF, database set-up and management, data entry and verification, data validation, QC of the database, and documentation of the performed activities including information of discrepancies in the process. Data validation/data cleaning procedures are designed to assure validity and accuracy of clinical data and consist of computerised online edit checks such as range checks and batch checks on data exports. All study-specific and standard data validation programming will be tested in a separate testing environment prior to use on production data. Detailed information on data management is described in a study-specific Data Management Plan (DMP).

Clinical data will be entered into an eCRF provided by Viedoc Technologies AB. Clinical data will be entered directly from the source documents or at bedside by authorised site

For peer review only - http://bmjopen.bmj.com/site/about/guidelines.xhtml

personnel designated by the Investigator. All entries of data, corrections, and alterations in the eCRF are to be made by the Investigator or designee.

Monitors will review the eCRFs for completeness and consistency and compare data with the respective source documents. If corrections are needed, queries will be raised within the eCRF, either as a result of built-in edit checks or manually and corrected by the site staff. When all data have been entered and discrepancies solved, clean file will be declared, the database will be locked, the code will be broken and the data will be analysed.

Statistical analysis plan

Continuous data will be presented in terms of evaluable and missing observations, arithmetic mean, standard deviation (SD), median, minimum, and maximum value. Categorical data will be presented as counts and percentages. When applicable, summary data will be presented by treatment, and by assessment time. Individual subject data will be listed by subject number, treatment, and, where applicable, by assessment time. All descriptive summaries and statistical analyses will be performed using SAS Version 9.4 or later (SAS Institute, Inc.). Baseline will be defined as the last non-missing observation prior to the first administration of the intervention.

Outliers will be included in summary tables and listings and will not be handled separately in any analyses. Generally, no imputation of missing data will be performed. However, clinical safety laboratory parameters that are outside the detection limit, will be replaced with the detection limit when calculating statistics. Also, when calculating statistics for pharmacokinetics plasma concentrations, concentrations under LLOQ will be replaced with LLOQ if more than 50% of the values for a given time point is above LLOQ. Otherwise, no statistics will be calculated for that time point.

Data Monitoring

Internal safety review committee (iSRC)

Before initiating a new dose group, all subjects in the previous dose group must have been treated and safety, tolerability, and pharmacokinetics data (at least data up to 5 days for pharmacokinetics and 8 days for safety) for all treated subjects must have been evaluated by the iSRC. In case of non-replaced dropouts, available data for the dropouts will be included in the iSRC evaluation.

For peer review only - http://bmjopen.bmj.com/site/about/guidelines.xhtml

If TCP-25 is considered safe and tolerable, a written recommendation on the next dose level will be provided to the Sponsor.

The voting members of the iSRC will consist of the principal investigator or delegate and the chief physician at the Clinical Trial Unit or delegate. In addition, the study clinical research manager, the study pharmacokineticist, and additional Sponsor representatives may be invited as appropriate. Further internal or external experts may be invited and consulted by the iSRC as appropriate.

The recommendation to the Sponsor may be study progression, to continue with a higher or lower dose than the intended dose, to repeat the same dose level, to continue with an intermediate dose level, or to stop dosing. The recommendation of the iSRC on the next dose level will be made in consensus between the iSRC members and documented as appropriate.

In case there is disagreement between the 2 voting members, the most conservative approach will be taken.

Auditing

The sponsor has employed a contract research organisation (Clinical Trial Consultants AB) as an independent monitor. Monitoring will be done according to a risk-based monitoring plan and the responsible monitor will periodically visit the study site at times agreed upon by the investigator and the monitor. At each monitoring visit, the role of the Monitor includes, but is not limited to:

- confirm that facilities and resources remain acceptable.
- confirm that the investigational team is adhering to the study protocol, applicable standard operating procedures, guidelines, manuals, and regulatory requirements.
- verify that data are being recorded in the eCRFs in an accurate and timely manner and that accountability checks are being performed.
- verify that data in the eCRF are consistent with the clinical records in accordance with the monitoring plan.
- verify that the correct informed consent procedure has been adhered to for participating subjects.

For peer review only - http://bmjopen.bmj.com/site/about/guidelines.xhtml

- verify that AEs are recorded and reported in a timely manner and according to the CSP.
- raise and escalate any serious quality issues, serious GCP breach and any data privacy breach to the Sponsor.

Centralised monitoring will also be performed continuously by study team members at CTC in accordance with the monitoring plan. When the study has been completed and all queries have been resolved and the database has been locked, the Monitor will perform a close-out visit.

Authorised representatives of the Sponsor, a competent authority, or an IEC may also perform audits or inspections at the research clinic, including source data verification.

Ethics and dissemination:

Ethical conduct of the study

The study will be performed in accordance with ethical principles that have their origin in the Declaration of Helsinki and are consistent with ICH/GCPE6 (R2), EU Clinical Trials Directive, and applicable local regulatory requirements. The principal investigator is responsible for submitting the study protocol, the subject information and informed consent form, any other written information to be provided to the subjects, and any advertisements used for subject recruitment to the Swedish Ethical Review Authority for approval. The study has been approved by the Swedish Ethical Review Authority (registration number 2022-00527-01)

Before performing any study-related procedures the informed consent form shall be signed and personally dated by the subject and by the investigator. The subjects will be covered under the sponsors liability insurance policy. The participating subjects are also protected in accordance with national regulations, as applicable.

Subject data protection

The informed consent form includes information that data will be recorded, collected, and processed and may be transferred to other European Economic Area (EEA) or non-EEA countries. In accordance with the General Data Protection Regulation (GDPR) (EU) 2016/679, the data will not identify any persons taking part in the study.

For peer review only - http://bmjopen.bmj.com/site/about/guidelines.xhtml

Clinical study report

A summarising report shall be submitted to the Swedish competent authority and IEC within 12 months after completion of the study. A clinical study report, in compliance with ICH-E3, describing the conduct of the study, any statistical analyses performed, and the results obtained, will be prepared. The report will be reviewed and approved by, as a minimum, the principal investigator, the statistician, and the sponsor. The study results will be reported in the EudraCT database per applicable regulations within 12 months after completion of the study. All data obtained from any exploratory analyses will be reported separately.

Publication and dissemination

Publication of the study results and access to the study data is at the discretion of the Sponsor. Authorship for any publications will be according to the Vancouver criteria. Subjects will be asked to provide a srelease for use of their photographs in publications if applicable. The subject has a right to refuse the photo release without jeopardising their eligibility to participate in the study.

Patient and public involvement

Patients or the public were neither involved in the design nor will they be involved in this clinical trial's conduct, reporting or dissemination plans.

Protocol version: Protocol version 2.0; 25 January 2022. Protocol amendments will be promptly communicated to all relevant parties, trial registries, and to the ethics committee and competent authority as required by law.

Acknowledgments: We acknowledge the support of the staff at the Clinical Trial Unit at Skane University Hospital Lund and Clinical Trial Consultants AB for input on the Clinical Study Protocol. Xinnate AB provided the project management resources and additional expertise for the regulatory development of TCP-25 gel and the completion of the Clinical Study Protocol. We thank Anne Nielsen and other personnel at the Department of Dermatology Lund and the Clinical Trial Unit at Skane University Hospital Lund for support with the clinical parts of the study. We used the SPIRIT checklist when writing this report (12).

For peer review only - http://bmjopen.bmj.com/site/about/guidelines.xhtml

Authors' contributions: SL, KW, prepared the first draft of the manuscript. AS and KS were responsible for conception of the study and contributed to the development of the clinical study protocol together with other co-authors. All authors revised and approved the final manuscript.

Roles and responsibilities: A contract research organization (Clinical Trial Consultants AB) will execute the study. the principal investigator and iSRC will carry out analysis, and interpretation of the data. The study sponsor, Xinnate AB, has ultimate responsibility for design of this study, its execution, analyses, interpretation of the data, and decision to submit results.

Funding: The Safety study described here was funded by Xinnate AB. Exploratory analyses on data and materials originating from this study will be integrated with other research projects funded by research grants from the Swedish Research Council (project 2020-02016), Edvard Welanders Stiftelse and Finsenstiftelsen (Hudfonden), the Österlund Foundation, and the Swedish Government Funds for Clinical Research (ALF).

Competing interests: AS is a co-founder and shareholder of Xinnate AB. The other authors report no competing interests.

For peer review only

Figure legends and tables

Figure 1. Overview of the study design. Wound photography, wound swab/filter, and dressing collection are samples to be collected and biobanked for future exploratory studies. IMP = investigational medicinal product (TCP-25); AE = adverse events; ECG = electrocardiogram

Table 1. Trial registration data

Data category	Information
**Primary registry and trial identifying number**	ClinicalTrials.gov (release pending)
**Date of registration in primary registry**	Release pending
**Secondary identifying numbers**	EudraCT 2021-004728-14
**Source(s) of monetary or material support**	Xinnate AB,
**Primary sponsor**	Xinnate AB
**Secondary sponsors**	None
**Contact for public queries**	Karim Saleh, MD, PhD<br>Email: karim.saleh@med.lu.se
**Contact for scientific queries**	Karim Saleh, MD, PhD (principal investigator)<br>Email: karim.saleh@med.lu.se
**Public title**	Safety, Tolerability, and Pharmacokinetics of Ascending Topical Doses of TCP-25 Applied to Epidermal Suction Blister Wounds
**Scientific title**	A Phase 1, Randomized, Double-Blind, Placebo-Controlled Study in Healthy Male and Female Volunteers to Investigate the Safety, Tolerability, and Pharmacokinetics of Ascending Topical Doses of TCP-25 Applied to Epidermal Suction Blister Wounds
**Countries of recruitment**	Sweden
**Health condition(s) or problem(s) studied**	Acute epidermal wounds
**Intervention(s)**	Active comparator: TCP-25 gel (0.86, 2.9, or 8.6 mg/mL)
	Placebo comparator: placebo gel (equivalent gel with no TCP-25)

For peer review only - http://bmjopen.bmj.com/site/about/guidelines.xhtml

Data category	Information
**Key inclusion and exclusion criteria**	Ages eligible for study: 18-60 years<br>Sexes eligible for study: both<br>Accepts healthy volunteers: yes
	Inclusion criteria: See table 2
	Exclusion criteria: See table 2
**Study type**	Interventional
	Allocation: randomized<br>Model: Sequential assignment (ascending doses)<br>Masking: Quadruple (Participant, Care Provider, Investigator, Outcomes Assessor)
	Primary purpose: Treatment
	Phase I
**Date of first enrolment**	April 7, 2022
**Target sample size**	24
**Recruitment status**	Recruiting
**Primary outcome(s)**	Adverse Events [Time Frame: 15 days]<br>Incidence of abnormal local reactions (Local tolerability) [Time Frame: Day 1, 2, 3, 5, 8, 11]<br>Number of patients with clinically significant changes from baseline in electrocardiogram (ECG), vital signs, and physical examinations [Time Frame: During screening (baseline) and on day 11]<br>Number of patients with clinically significant changes from baseline in safety laboratory parameters [Time Frame: During screening (baseline) and on day 2, 3, 5, 11]
**Key secondary outcomes**	Plasma concentration of TCP-25 [Time Frame: Day 1 (measured before blister formation), Day 2 (measured before administration of the intervention and 0.5 and 1 hours after administration of the intervention), Day 3 (measured before administration of the intervention 1 hour after administration of the intervention), and Day 5 (measured before administration of intervention)
**Ethics review**	Approved 2022 March 10, Eti kprövningsmyndigheten registration number 2022-00527-01

For peer review only - http://bmjopen.bmj.com/site/about/guidelines.xhtml

1 **Table 2. Summary of inclusion and exclusion criteria**

Inclusion criteria	Exclusion criteria
1. Willing and able to give written informed consent for participation in the study.<br>2. Healthy male or female subject 18-60 years (inclusive) of age at the time of signing the informed consent.<br>3. Body Mass Index (BMI) ≥ 18.0 and ≤ 30.0 kg/m2.<br>4. Healthy and intact skin where the blister suction wounds will be induced.<br>5. Women of childbearing potential must have a documented negative serum pregnancy test done at the screening visit, within 4 weeks prior to suction blister formation and the start of study treatment.<br>Male subjects must be willing to use condom or be vasectomised or practice sexual abstinence to prevent pregnancy.<br>6. Clinically relevant medical history, physical findings, vital signs, ECG and laboratory values at the time of screening.	1. History of any clinically significant disease or disorder.<br>2. Any disease that may interfere with wound healing.<br>3. Active skin disease and/or tattoos in the areas where suction blister wounds will be induced.<br>4. Any planned major surgery within the duration of the study.<br>5. Any vital sign values outside the normal range.<br>6. Any clinically significant abnormalities in the resting ECG at the time of screening.<br>7. Current smokers or users of nicotine products.<br>8. Female subjects who are pregnant or lactating or planning a pregnancy.<br>9. Systemic immunosuppressive treatment<br>10. Subjects who are currently receiving or have received antibiotics or systemic corticosteroids within 2 weeks prior to screening.<br>11. Regular use of anticoagulants or non-steroidal anti-inflammatory drugs (NSAIDs) within 2 weeks prior to the (first) administration of the intervention.

For peer review only - http://bmjopen.bmj.com/site/about/guidelines.xhtml

1 **Table 3. Safety laboratory parameters**

Category	Parameter
**Clinical chemistry**	Alanine aminotransferase (ALT)
	Aspartate aminotransferase (AST)
	Creatinine
	C-reactive protein (CRP)
	Glucose
	HbA1C
**Haematology**	Haematocrit
	Haemoglobin (Hb)
	Erythrocytes
	Mean corpuscular volume (MCV)
	Mean corpuscular haemoglobin (MCH)
	Mean corpuscular haemoglobin concentration (MCHC)
	Thrombocytes
	Leukocytes
	Eosinophils
	Neutrophils
	Basophils
	Lymphocytes
	Monocytes
**Coagulation**	Activated partial thromboplastin time (APTT)<br>Prothrombin complex/International Normalised Ratio (PK/INR)
**Pregnancy test (WOCBP only)**	Serum pregnancy test at screening
	Urine pregnancy test (Visit 2)

2

3

4

For peer review only - http://bmjopen.bmj.com/site/about/guidelines.xhtml

References

1. Eming SA, Krieg T, Davidson JM. Inflammation in Wound Repair: Molecular and Cellular Mechanisms. Journal of Investigative Dermatology. 2007;127(3):514-25.

2. Sen CK, Gordillo GM, Roy S, Kirsner R, Lambert L, Hunt TK, et al. Human skin wounds: a major and snowballing threat to public health and the economy. Wound Repair Regen. 2009;17(6):763-71.

3. Nathan C, Cars O. Antibiotic Resistance — Problems, Progress, and Prospects. New England Journal of Medicine. 2014;371(19):1761-3.

4. Filius PMG, Gyssens IC. Impact of Increasing Antimicrobial Resistance on Wound Management. American Journal of Clinical Dermatology. 2002;3(1):1-7.

5. Michael CA, Dominey-Howes D, Labbate M. The antimicrobial resistance crisis: causes, consequences, and management. Front Public Health. 2014;2:145.

6. Puthia M, Butrym M, Petrlova J, Strömdahl AC, Andersson M, Kjellström S, et al. A dual-action peptide-containing hydrogel targets wound infection and inflammation. Sci Transl Med. 2020;12(524).

7. Strömdahl AC, Ignatowicz L, Petruk G, Butrym M, Wasserstrom S, Schmidtchen A, et al. Peptide-coated polyurethane material reduces wound infection and inflammation. Acta Biomater. 2021;128:314-31.

8. Kottner J, Hillmann K, Fimmel S, Seité S, Blume-Peytavi U. Characterisation of epidermal regeneration in vivo: a 60-day follow-up study. J Wound Care. 2013;22(8):395-400.

9. Ahlström MG, Gjerdrum LMR, Larsen HF, Fuchs C, Sørensen AL, Forman JL, et al. Suction blister lesions and epithelialization monitored by optical coherence tomography. Skin Res Technol. 2018;24(1):65-72.

10. Daeschlein G, Alborova J, Patzelt A, Kramer A, Lademann J. Kinetics of physiological skin flora in a suction blister wound model on healthy subjects after treatment with water-filtered infrared-A radiation. Skin Pharmacol Physiol. 2012;25(2):73-7.

11. Larsen HF, Ahlström MG, Gjerdrum LMR, Mogensen M, Ghathian K, Calum H, et al. Noninvasive measurement of reepithelialization and microvascularity of suction-blister wounds with benchmarking to histology. Wound Repair Regen. 2017;25(6):984-93.

12. Chan A-W, Tetzlaff JM, Gøtzsche PC, Altman DG, Mann H, Berlin JA, et al. SPIRIT 2013 explanation and elaboration: guidance for protocols of clinical trials. BMJ : British Medical Journal. 2013;346:e7586.

For peer review only

For peer review only - http://bmjopen.bmj.com/site/about/guidelines.xhtml

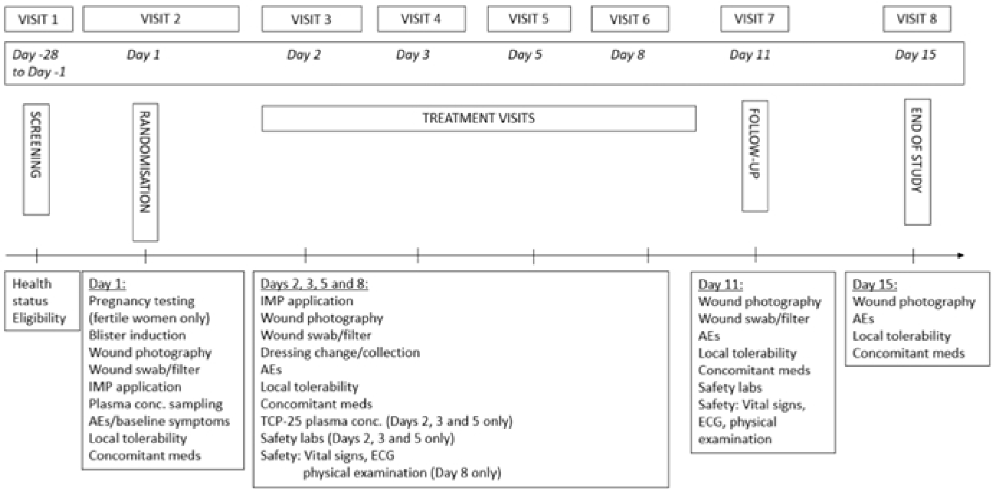

Figure 1. Overview of the study design. Wound photography, wound swab/filter, and dressing collection are samples to be collected and biobanked for future exploratory studies. IMP = investigational medicinal product (TCP-25); AE = adverse events; ECG = electrocardiogram

464x230mm (38 x 38 DPI)

Supplement 1, Full inclusion and exclusion criteria

Inclusion criteria	Exclusion criteria
1. Willing and able to give written informed consent for participation in the study.	1. History of any clinically significant disease or disorder which, in the opinion of the Investigator, may either put the subject at risk because of participation in the study, or influence the results or the subject's ability to participate in the study.
2. Healthy male or female subject 18-60 years (inclusive) of age at the time of signing the informed consent.	2. Disease that may interfere with wound healing, e.g., diabetes type I/II, arterial-, renal-, liver, or cardiac insufficiency, chronic obstructive lung disease, cancer, autoimmune disease, edema at the study site, severe obesity, or previous known wound healing problems, as judged by the investigator.
3. Body Mass Index (BMI) ≥ 18.0 and ≤ 30.0 kg/m2.	3. Active skin disease, e.g., dermatitis, psoriasis and wounds, and/or tattoos in the areas where suction blister wounds will be induced, as judged by the investigator.
4. Healthy and intact skin where the blister suction wounds will be induced.	4. Any planned major surgery within the duration of the study.
5. Women of childbearing potential (WOCBP) must have a documented negative serum pregnancy test done at the screening visit, within 4 weeks prior to suction blister formation and the start of study treatment.<br>WOCBP must practice abstinence (only allowed when this is the preferred and usual lifestyle of the subject) or must agree to use a highly effective method of contraception with a failure rate of < 1% to prevent pregnancy<br>Male subjects must be willing to use condom or be vasectomised or practice sexual abstinence to prevent pregnancy and drug exposure of a partner and refrain from donating sperm from the date of last dosing until 3 months after the last dosing with the IMP. Their female partner of child-bearing potential must use contraceptive methods with a failure rate of < 1% to prevent pregnancy (see above).	5. After 10 minutes supine rest at the time of screening, any vital signs values outside the following ranges:<br>• Systolic blood pressure 160 mmHg, or<br>• Diastolic blood pressure 100 mmHg, or<br>• Pulse 90 beats per minute (bpm)
6. Clinically relevant medical history, physical findings, vital signs, ECG and laboratory values at the time of screening, as judged by the Investigator.	6. Any clinically significant abnormalities in the resting ECG at the time of screening, as judged by the Investigator.
	7. Current smokers or users of nicotine products. Irregular use of nicotine (e.g., smoking, snuffing, chewing tobacco) less than three times per week is allowed before screening visit.
	8. Female subjects who are pregnant or lactating or planning a pregnancy.
	9. Systemic immunosuppressive treatment
	10. Subjects who are currently receiving or have received the following treatments within 2 weeks prior to screening are excluded from the

For peer review only

	study: - systemic corticosteroids or immunosuppressant agents; or - antibiotics via any route
	11. Regular use of anticoagulants (i.e., heparin, warfarin, coumarins, other anticoagulants per Investigator's judgement) or non-steroidal anti-inflammatory drugs (NSAIDs) within 2 weeks prior to the (first) administration of IMP, at the discretion of the Investigator.
	12. History of severe allergy/hypersensitivity or ongoing allergy/hypersensitivity, as judged by the Investigator, or history of hypersensitivity to drugs with a similar chemical structure or class to TCP-25 or to any excipients of the hydrogel.
	13. Planned treatment or treatment with another investigational drug within 3 months prior to Day -1.
	14. History of alcohol abuse or excessive intake of alcohol, as judged by the Investigator.
	15. Presence or history of drug abuse, as judged by the Investigator
	16. Plasma donation within one month of screening or blood donation (or corresponding blood loss) during the three months prior to screening.
	17. Involvement in the planning and/or conduct of the study.
	18. Investigator considers the subject unlikely to comply with study procedures, restrictions and requirements.

For peer review only

Supplement 2, Restrictions during the study

- Contraception Requirements: The male volunteers are expected to use condom to prevent pregnancy and drug exposure of a partners and refrain from donating sperm from the date of dosing until 4 weeks after the last dosing of the IMP.
  Fertile female study subjects are expected to practice abstinence or use contraceptive methods with a failure rate of < 1% to prevent pregnancy during the same period, i.e., combined [oestrogen and progestogen containing] hormonal contraception associated with inhibition of ovulation [oral, intravaginal, transdermal], progestogen-only hormonal contraception associated with inhibition of ovulation [oral, injectable, implantable], IUD or intrauterine IUS) from at least 4 weeks prior to dose to 4 weeks after last dose.
- Caffeine: Consumption of caffeine equivalent to up to 5 cups of coffee per day will be allowed from screening to the end-of-study visit.
- Other xanthine or taurine containing products/beverages than tea or coffee: Energy drinks (e.g., Red Bull, Battery, Flying Horse, La Bomba) are not allowed from screening to the end-of-study visit.
- Nicotine: Smoking (>3 cigarettes per day) or use of nicotine-containing products is not allowed from screening to the end-of-study visit.
- Exercise: From Day 1 to Day 11, the subjects must abstain from strenuous exercise that would cause excessive sweating compromising the dressing of the wounds.
- Blood donation: The subjects must not donate blood or plasma during the study until 3 months after the final medical examination at the end-of-study visit.
- Participation in other clinical studies: Study subjects are not allowed to participate in any other interventional clinical study during the study period.

Supplement 3, Prior and concomitant theraphy

Prohibited medication

From 2 weeks prior to wounding and throughout the study until Visit 9 (unless indicated for treatment of any AEs):

- Systemic corticosteroids or immunosuppressant agents; or
- Antibiotics via any route
- Anticoagulants (e.g., heparin, warfarin, coumarins, other anticoagulants per Investigator's judgement)
- Regular use of NSAIDs (i.e., ibuprofen, diclofenac, acetylsalicylic acid and others)

Allowed medication

- Paracetamol 1 g up to 4 times daily is allowed for treatment of post-procedural pain.
- Any medications at a stable dose to treat medical history events will be allowed throughout the study.
- Medications considered necessary for the subject´s safety and wellbeing may be given at the discretion of the Investigator during the study. Following consultation with the Sponsor, the Investigator will determine whether or not the subject should continue in the study.

For peer review only

Supplement 4, General withdrawal criteria

Subjects are free to discontinue their participation in the study at any time and for whatever reason without affecting their right to an appropriate follow-up investigation or their future care. If possible, the reason for withdrawal of consent should be documented.

Subjects may be discontinued from the study at any time at the discretion of the Investigator.

Reasons for discontinuation include:

- Subject decision
- Severe non-compliance to study protocol procedures, as judged by the Investigator and/or Sponsor
- Subject is lost to follow-up.
- Significant AEs posing a risk for the subject, as judged by the Investigator and/or Sponsor
- Withdrawal of informed consent to the use of biological samples
- Pregnancy
- Death
- Meeting of an exclusion criterion during the study, which, in the opinion of the Investigator, may pose a risk for the subject
- Use of prohibited medication

For peer review only

Reporting checklist for protocol of a clinical trial.

Based on the SPIRIT guidelines.

Instructions to authors

Complete this checklist by entering the page numbers from your manuscript where readers will find each of the items listed below.

Your article may not currently address all the items on the checklist. Please modify your text to include the missing information. If you are certain that an item does not apply, please write "n/a" and provide a short explanation.

Upload your completed checklist as an extra file when you submit to a journal.

In your methods section, say that you used the SPIRITreporting guidelines, and cite them as:

Chan A-W, Tetzlaff JM, Gøtzsche PC, Altman DG, Mann H, Berlin J, Dickersin K, Hróbjartsson A, Schulz KF, Parulekar WR, Krleža-Jerić K, Laupacis A, Moher D. SPIRIT 2013 Explanation and Elaboration: Guidance for protocols of clinical trials. BMJ. 2013;346:e7586

		Reporting Item	Page Number
**Administrative information**			
Title	#1	Descriptive title identifying the study design, population, interventions, and, if applicable, trial acronym	Page 1
Trial registration	#2a	Trial identifier and registry name. If not yet	Page 2

For peer review only

		registered, name of intended registry	
Trial registration: data set	#2b	All items from the World Health Organization Trial Registration Data Set	Table 1
Protocol version	#3	Date and version identifier	Page 13
Funding	#4	Sources and types of financial, material, and other support	Page 13
Roles and responsibilities: contributorship	#5a	Names, affiliations, and roles of protocol contributors	Page 14
Roles and responsibilities: sponsor contact information	#5b	Name and contact information for the trial sponsor	Page 13
Roles and responsibilities: sponsor and funder	#5c	Role of study sponsor and funders, if any, in study design; collection, management, analysis, and interpretation of data; writing of the report; and the decision to submit the report for publication, including whether they will have ultimate authority over any of these activities	Page 13
Roles and responsibilities: committees	#5d	Composition, roles, and responsibilities of the coordinating centre, steering committee, endpoint adjudication committee, data	Page 10-11, Page 13

For peer review only

		management team, and other individuals or groups overseeing the trial, if applicable (see Item 21a for data monitoring committee)	
**Introduction**			
Background and rationale	#6a	Description of research question and justification for undertaking the trial, including summary of relevant studies (published and unpublished) examining benefits and harms for each intervention	Page 3
Background and rationale: choice of comparators	#6b	Explanation for choice of comparators	Page 4
Objectives	#7	Specific objectives or hypotheses	Page 4
Trial design	#8	Description of trial design including type of trial (eg, parallel group, crossover, factorial, single group), allocation ratio, and framework (eg, superiority, equivalence, non-inferiority, exploratory)	Page 4-5
**Methods: Participants, interventions, and outcomes**			
Study setting	#9	Description of study settings (eg, community	Page 4-5

For peer review only

		clinic, academic hospital) and list of countries where data will be collected. Reference to where list of study sites can be obtained	
Eligibility criteria	#10	Inclusion and exclusion criteria for participants. If applicable, eligibility criteria for study centres and individuals who will perform the interventions (eg, surgeons, psychotherapists)	Page 5
Interventions: description	#11a	Interventions for each group with sufficient detail to allow replication, including how and when they will be administered	Page 6
Interventions: modifications	#11b	Criteria for discontinuing or modifying allocated interventions for a given trial participant (eg, drug dose change in response to harms, participant request, or improving / worsening disease)	Page 5
Interventions: adherance	#11c	Strategies to improve adherence to intervention protocols, and any procedures for monitoring adherence (eg, drug tablet return; laboratory tests)	N/A (all study intervention protocols will be carried out by a study nurse, so patient compliance is not a factor).
Interventions: concomitant care	#11d	Relevant concomitant care and interventions that are permitted or prohibited during the trial	Page 6, supplement 2 and 3

For peer review only

Outcomes	#12	Primary, secondary, and other outcomes, including the specific measurement variable (eg, systolic blood pressure), analysis metric (eg, change from baseline, final value, time to event), method of aggregation (eg, median, proportion), and time point for each outcome. Explanation of the clinical relevance of chosen efficacy and harm outcomes is strongly recommended	Page 6-7
Participant timeline	#13	Time schedule of enrolment, interventions (including any run-ins and washouts), assessments, and visits for participants. A schematic diagram is highly recommended (see Figure)	Figure 1
Sample size	#14	Estimated number of participants needed to achieve study objectives and how it was determined, including clinical and statistical assumptions supporting any sample size calculations	Page 8
Recruitment	#15	Strategies for achieving adequate participant enrolment to reach target sample size	Page 8

Methods: Assignment of interventions (for

For peer review only

controlled trials)

Allocation: sequence generation	#16a	Method of generating the allocation sequence (eg, computer-generated random numbers), and list of any factors for stratification. To reduce predictability of a random sequence, details of any planned restriction (eg, blocking) should be provided in a separate document that is unavailable to those who enrol participants or assign interventions	Page 8
Allocation concealment mechanism	#16b	Mechanism of implementing the allocation sequence (eg, central telephone; sequentially numbered, opaque, sealed envelopes), describing any steps to conceal the sequence until interventions are assigned	Page 8
Allocation: implementation	#16c	Who will generate the allocation sequence, who will enrol participants, and who will assign participants to interventions	Page 8
Blinding (masking)	#17a	Who will be blinded after assignment to interventions (eg, trial participants, care providers, outcome assessors, data analysts), and how	Page 9
Blinding (masking): emergency unblinding	#17b	If blinded, circumstances under which unblinding is permissible, and procedure for revealing a participant's allocated intervention	Page 9

For peer review only

		during the trial	
**Methods: Data collection, management, and analysis**			
Data collection plan	#18a	Plans for assessment and collection of outcome, baseline, and other trial data, including any related processes to promote data quality (eg, duplicate measurements, training of assessors) and a description of study instruments (eg, questionnaires, laboratory tests) along with their reliability and validity, if known. Reference to where data collection forms can be found, if not in the protocol	Page 7 - 8
Data collection plan: retention	#18b	Plans to promote participant retention and complete follow-up, including list of any outcome data to be collected for participants who discontinue or deviate from intervention protocols	Page 5, 6
Data management	#19	Plans for data entry, coding, security, and storage, including any related processes to promote data quality (eg, double data entry; range checks for data values). Reference to where details of data management	Page 9

For peer review only

		procedures can be found, if not in the protocol	
Statistics: outcomes	#20a	Statistical methods for analysing primary and secondary outcomes. Reference to where other details of the statistical analysis plan can be found, if not in the protocol	Page 10
Statistics: additional analyses	#20b	Methods for any additional analyses (eg, subgroup and adjusted analyses)	Page 10
Statistics: analysis population and missing data	#20c	Definition of analysis population relating to protocol non-adherence (eg, as randomised analysis), and any statistical methods to handle missing data (eg, multiple imputation)	Page 10
**Methods: Monitoring**			
Data monitoring: formal committee	#21a	Composition of data monitoring committee (DMC); summary of its role and reporting structure; statement of whether it is independent from the sponsor and competing interests; and reference to where further details about its charter can be found, if not in the protocol. Alternatively, an explanation of why a DMC is not needed	Page 10 - 11
Data monitoring: interim analysis	#21b	Description of any interim analyses and stopping guidelines, including who will have access to these interim results and make the	Page 10-11

For peer review only

		final decision to terminate the trial	
Harms	#22	Plans for collecting, assessing, reporting, and managing solicited and spontaneously reported adverse events and other unintended effects of trial interventions or trial conduct	Page 7-8
Auditing	#23	Frequency and procedures for auditing trial conduct, if any, and whether the process will be independent from investigators and the sponsor	Page 11-12
**Ethics and dissemination**			
Research ethics approval	#24	Plans for seeking research ethics committee / institutional review board (REC / IRB) approval	Page 12
Protocol amendments	#25	Plans for communicating important protocol modifications (eg, changes to eligibility criteria, outcomes, analyses) to relevant parties (eg, investigators, REC / IRBs, trial participants, trial registries, journals, regulators)	Page 13
Consent or assent	#26a	Who will obtain informed consent or assent from potential trial participants or authorised surrogates, and how (see Item 32)	Page 5

For peer review only

Consent or assent: ancillary studies	#26b	Additional consent provisions for collection and use of participant data and biological specimens in ancillary studies, if applicable	Page 5
Confidentiality	#27	How personal information about potential and enrolled participants will be collected, shared, and maintained in order to protect confidentiality before, during, and after the trial	Page 12
Declaration of interests	#28	Financial and other competing interests for principal investigators for the overall trial and each study site	Page 14
Data access	#29	Statement of who will have access to the final trial dataset, and disclosure of contractual agreements that limit such access for investigators	Page 13
Ancillary and post trial care	#30	Provisions, if any, for ancillary and post-trial care, and for compensation to those who suffer harm from trial participation	Ancillary and post-trial care: not applicable. Compensation (insurance): Page 12
Dissemination policy: trial results	#31a	Plans for investigators and sponsor to communicate trial results to participants, healthcare professionals, the public, and other relevant groups (eg, via publication, reporting in results databases, or other data sharing	Page 13

For peer review only

		arrangements), including any publication restrictions	
Dissemination policy: authorship	#31b	Authorship eligibility guidelines and any intended use of professional writers	Page 13
Dissemination policy: reproducible research	#31c	Plans, if any, for granting public access to the full protocol, participant-level dataset, and statistical code	N/A (no plans)

Appendices

Informed consent materials	#32	Model consent form and other related documentation given to participants and authorised surrogates	N/A (not included for publication)
Biological specimens	#33	Plans for collection, laboratory evaluation, and storage of biological specimens for genetic or molecular analysis in the current trial and for future use in ancillary studies, if applicable	Page 4

Notes:

The SPIRIT Explanation and Elaboration paper is distributed under the terms of the Creative Commons Attribution License CC-BY-NC. This checklist was completed on 16. May 2022 using https://www.goodreports.org/, a tool made by the EQUATOR Network in collaboration with Penelope.ai

For peer review only

BMJ Open

Study protocol for a Phase 1, Randomized, Double-Blind, Placebo-Controlled Study to Investigate the Safety, Tolerability, and Pharmacokinetics of Ascending Topical Doses of TCP-25 Applied to Epidermal Suction Blister Wounds in Healthy Male and Female Volunteers

Journal:	*BMJ Open*
Manuscript ID	bmjopen-2022-064866.R1
Article Type:	Protocol
Date Submitted by the Author:	08-Dec-2022
Complete List of Authors:	Lundgren, Sigrid; Lund University, Department of Clinical Sciences Lund; Skåne University Hospital Lund, Department of Dermatology<br>Wallblom, Karl; Lund University, Department of Clinical Sciences Lund; Skåne University Hospital Lund, Department of Dermatology<br>Fisher, Jane; Lund University, Department of Clinical Sciences Lund<br>Erdmann, Susanne; Skåne University Hospital Lund, Department of Dermatology<br>Smidtchen, Artur; Lund University, Department of Clinical Sciences Lund; Skåne University Hospital Lund, Department of Dermatology<br>Saleh, Karim; Lund University, Department of Clinical Sciences Lund; Skåne University Hospital Lund, Department of Dermatology
**Primary Subject Heading**:	Dermatology
Secondary Subject Heading:	Dermatology
Keywords:	WOUND MANAGEMENT, Clinical trials < THERAPEUTICS, DERMATOLOGY

SCHOLARONE™
Manuscripts

Study protocol for a Phase 1, Randomized, Double-Blind, Placebo-Controlled Study to Investigate the Safety, Tolerability, and Pharmacokinetics of Ascending Topical Doses of TCP-25 Applied to Epidermal Suction Blister Wounds in Healthy Male and Female Volunteers

Sigrid Lundgren[1, 2*a], Karl Wallblom[1, 2*], Jane Fisher[1], Susanne Erdmann[2], Artur Schmidtchen[1, 2, 3], Karim Saleh[1, 2].

Affiliations:

[1] Section for Dermatology and Venereology, Department of Clinical Sciences Lund, Lund University, Lund, Sweden

[2] Department of Dermatology, Skane University Hospital, Lund, Sweden

[3] Copenhagen Wound Healing Center, Bispebjerg Hospital, Department of Biomedical Sciences, University of Copenhagen, Copenhagen, Denmark

* Equal contribution

[a] Corresponding author

Email addresses:

Sigrid Lundgren: sigrid.lundgren@med.lu.se

Karl Wallblom: karl.wallblom@med.lu.se

Jane Fisher: jane.fisher@med.lu.se

Susanne Erdmann: susanne.erdman@banhof.se

Artur Schmidtchen: artur.schmidtchen@med.lu.se

Karim Saleh: karim.saleh@med.lu.se

Word Count: 4872 words

Key words: Wounds and injuries; Wound infection; Wound Healing

For peer review only

For peer review only - http://bmjopen.bmj.com/site/about/guidelines.xhtml

Abstract

Introduction

TCP-25 gel is intended for use in treatment of wound infection and inflammation. Current local therapies for wounds have limited efficacy to prevent infections and there are no wound treatments available today that target the excessive inflammation that often hampers wound healing in both acute and chronic wounds. There is therefore a high medical need for new therapeutic alternatives.

Methods and analysis

A randomised, double-blinded, first-in-human study was designed to evaluate the safety, tolerability, and potential systemic exposure of three increasing doses of the TCP-25 gel applied topically on suction blister wounds in healthy adults. The dose-escalation will be divided into 3 sequential dose groups with 8 subjects in each group (24 patients in total). Within each dose group, the subjects will receive four wounds, with two wounds on each thigh. Each subject will receive TCP-25 on one wound per thigh and placebo on one wound per thigh in a randomised double-blinded manner, with a reverse reciprocal position on each respective thigh, to a total of 5 doses over 8 days. An internal safety review committee will monitor emerging safety and plasma concentration data over the course of the study and must give a favourable recommendation prior to initiating the next dose group, which will receive placebo gel or a higher concentration of TCP-25 in exactly the same manner described above.

Ethics and dissemination

The study will be performed in accordance with ethical principles consistent with the Declaration of Helsinki, ICH/GCPE6 (R2), EU Clinical Trials Directive, and applicable local regulatory requirements.

This study is approved by the Swedish Medical Products Agency and the Swedish ethics committee under the registration number 2022-00527-01. The results of this study will be disseminated via publication to a peer-reviewed journal at the discretion of the sponsor.

Trial registration number: EudraCT 2021-004728-14, Clinical trials.gov NCT05378997

Strengths and limitations of this study

- Wound induction through the suction blister technique is minimally invasive and provides a consistent, though limited, wound area

For peer review only - http://bmjopen.bmj.com/site/about/guidelines.xhtml

For peer review only

- Before each dose increase, an internal safety review committee evaluates the emerging safety data, ensuring utmost attention to safety
- Frequent outcome assessment is a scientific strength, but could limit the study as it is time-consuming and burdensome for participants
- This is a single-centre study with a limited number of participants, in a specific age range, thus limiting its applicability to other populations

Introduction

In normal conditions, wounds heal in a sequenced and timely manner, characterised by four major phases (haemostasis, inflammation, proliferation, and remodelling). This is a complex process involving chemokines, growth factors, cytokines, proteases and antiproteases, and multiple cell types that all work in a controlled manner during the healing process. This timely process can be interrupted at various stages and by different factors, leading to a dysfunctional inflammatory phase and disrupting wound healing. This common feature characterises most major wound complications. (1)

Wounds of various types have a significant impact on patients, health care, and society (1). Types of wounds include acute post-surgical wounds and burns, and non-healing wounds resulting from diabetes or circulatory disturbances (2). It is critical both to prevent and treat infections and to address excessive inflammation in various wound types. To our knowledge there is currently no available wound treatment that targets both the infection and the excessive inflammation simultaneously. Furthermore, as antimicrobial resistance increases, common antibiotics and some antiseptics are becoming even less effective in treating wound infections. Antimicrobial resistant bacteria are often present in various wounds, thus increasing the risk of antimicrobial resistant infections (3-5). Therefore, there is a great need for new and cost-effective treatments that will improve healing and reduce both infection and inflammation in patients with various wounds.

TCP-25 is a new class of peptide-based drug which has a dual action targeting both infection and the associated excessive inflammation. A hydrogel containing TCP-25 treated infection and inflammation in murine and porcine wound infection models leading, hastening healing of infected wounds (6, 7). The mode of action is well characterised and involves interactions with bacterial lipopolysaccharide (LPS), other bacterial products, and the cell-receptor CD14, enabling a down-modulation of inflammation, while simultaneously exerting direct

For peer review only - http://bmjopen.bmj.com/site/about/guidelines.xhtml

antimicrobial effects. Importantly, TCP-25 acts on multiple multi-drug resistant bacterial isolates (6).

Suction blister wounds are standardised with respect to size, closure time, and wound depth, as the epidermal layer always is ablated at the same level, exposing the dermis (8). The mechanical nonbleeding excoriation elicits an innate immune response accompanied by increased blood flow and inflammatory cell infiltration in the underlying dermis. Epidermal regeneration occurs from the edges and appendages (9). Usually, the wounds are colonised by commensal bacteria after some days (10, 11).

The safety and tolerability of three dose levels of TCP-25 gel will be tested to monitor safety in experimental wounds generated by the suction blister technique. It will be compared to a placebo control gel which is identical to TCP-25 gel, without the active ingredient (TCP-25). An overview of trial registration data is summarized in **Table 1**.

Study objectives

The primary objective of this study is to evaluate the safety and tolerability of three dose levels of TCP-25 gel (0.86 mg/mL, 2.9 mg/mL and 8.6 mg/mL) applied topically to epidermal suction blister wounds. **The secondary objective** is to evaluate the systemic exposure of TCP-25 after topical application to epidermal suction blister wounds.

Exploratory objectives include evaluation of wounds from photographs, and to collect and store samples from dressings and wound surfaces for future exploratory research. The exploratory analyses will be described in a separate report.

Methods and analysis:

We used the SPIRIT checklist when writing this report (12).

Trial design and setting

This trial is a randomised, double-blinded, FIH study designed to evaluate the safety, tolerability, and potential systemic exposure of three increasing doses of the TCP-25 gel applied topically on suction blister wounds in healthy adults. The study will run as a single-centre study, conducted at Skåne University Hospital in Lund, Sweden. Screening and enrolment of the first dose group started on April 7, 2022. Randomization and treatment administration started on April 25, 2022. The final follow-up for the last participant in the initial target sample size (24 participants) is estimated to be at the end of June 2022, with the possibility of extension beyond this date to include more participants. Data analysis will begin in the April 2023. The dose-escalation will be divided into 3 sequential groups with 8 subjects

For peer review only - http://bmjopen.bmj.com/site/about/guidelines.xhtml

in each group. Within each dose group, each subject will receive TCP-25 on one wound on each thigh and placebo on one wound on each thigh in a randomised manner, but with a reverse reciprocal position on each respective thigh, to a total of 5 doses over 8 days. An internal safety review committee (iSRC) will monitor emerging safety and plasma concentration data over the course of the study and must give a favourable recommendation prior to initiating the higher dose in groups 2 and 3, based on safety parameters as described below. **Figure 1** presents an overview of the study design and which interventions and testing will be done on each day.

Eligibility criteria

Prior to enrolment in the study, all subjects will be screened. Subjects will only be included if they fulfil all of the inclusion criteria and none of the exclusion criteria. A summary of the eligibility criteria is listed in **Table 2**. Details of the eligibility criteria, prior and concomitant therapy restrictions, and general restrictions during the study can be found in **Supplements 1,2, and 3.**

Consent and withdrawal

All subjects will be provided with written and oral information about the trial, including collection of biological specimens for exploratory studies, by the investigator or authorized associate, and given sufficient time to make an informed decision about participation. The informed consent will be collected by a designated physician who is responsible for screening. Informed consent must be signed and documented before any study-specific procedures are performed.

Subjects will be free to discontinue their participation in the study at any time and for whatever reason without affecting their right to an appropriate follow-up investigation or their future care. If possible, the reason for withdrawal of consent shall be documented. Subjects may also be discontinued from the study at any time at the discretion of the Investigator according to the criteria described in **Supplement 4**. A subject who prematurely discontinues participation in the study will be asked about the presence of any adverse events and the Investigator must ask the subject if he/she is willing to be assessed according to the procedures scheduled for the end-of-study visit. Any ongoing adverse events will be followed until they are resolved.

For peer review only - http://bmjopen.bmj.com/site/about/guidelines.xhtml

Suction blister wound formation

Blister wounds will be formed on the medial aspect of the thigh, two blisters on each leg with a 6 cm distance in between them. Before wounding, hair at the wound sites will be shaved. Each wound site will be wiped with an ethanol-soaked gauze. The exact wound sites will be marked using a skin marker.

The blisters will be made using the Model NP-4 (Electronic Diversities, Finksburg, MD) suctioning device (13) operating in the negative pressure range 200-400 mmHg with heating for optimal blister formation. Blister wound diameter will be 10 mm. The device will run until all blisters have formed, which takes approximately 60-70 minutes. The roof of the blister will be excised using a sterile forceps and scissors.

The Model NP-4 suctioning device used in this study has the possibility to induce 4 wounds at the same time without causing notably more discomfort for the patient. We chose to make 4 wounds on each patient to increase amount of the safety data we will obtain, and therefore the rigour of the study, without requiring the inclusion of more patients.

Interventions

Treatment: Each wound will receive 0.15 mL of a sterile gel containing either TCP-25 (0.86, 2.9, or 8.6 mg/mL according to the dose group) or placebo (0 mg/mL TCP-25), applied topically.

TCP-25 is a synthetic peptide based on the 25 C-terminal amino acids of human thrombin, with a molecular weight of 3088.6 Da (6, 7). TCP-25 is soluble in water and formulated in a proprietary hydrogel based on hydroxyethyl cellulose with glycerol included for isotonicity. pH is 7.0.

When the three chosen concentrations (0.86 mg/mL, 2.9 mg/mL and 8.6 mg/mL) are applied in a volume of 0.15ml to a wound area of 0.8 cm^2, this results in total doses of 0.16mg/cm^2, 0.54mg/cm^2 and 1.6mg/ cm^2 respectively. These doses were based on effective doses identified in *in vitro* and *in vivo* tests (6, 7). Furthermore, the dose escalation steps were discussed with regulatory experts and selected to adequately assess safety and exploratory variables.

The pre-clinical safety of the drug product has been assessed through the necessary toxicity tests in mouse and porcine models, approved by the Swedish Medical Products Agency prior to initiation of this trial.

For peer review only - http://bmjopen.bmj.com/site/about/guidelines.xhtml

Dressing: After application of the assigned intervention, each wound will be dressed using a 2 x 2 cm polyurethane foam (Mepilex transfer, Mölnlycke health care) as primary dressing and covered with a secondary dressing (Tegaderm, 3M™). A secondary protective layer will be applied, consisting of overlapping gauze swabs covered with a secondary Tegaderm dressing.

Outcomes

The follow-up period will be 15 days after application of the intervention. This short time period ensures participant retention and complete follow-up. The exact time points for evaluation of the following outcomes can be seen in **Figure 1**. The methods for assessment are described under "data collection" below.

The primary objective of safety and tolerability will be evaluated using the following

Primary outcomes:

- Frequency, intensity, and seriousness of adverse events (AEs) on day 1-15.
- Local tolerability by direct investigator evaluation of the incidence of abnormal local reactions as compared to expected wound healing outcome on days 1, 2, 3, 5, 8, 11, and 15, as described in the data collection section below.
- Clinically significant changes from baseline, as determined by the investigators, in the following measures (measured at baseline and on day 11):
  - Electrocardiogram (ECG)
  - Vital signs (systolic/diastolic blood pressure, pulse rate)
  - Physical examinations (general condition, lymph nodes, throat, heart, lungs, and abdomen)
- Clinically significant changes from baseline, as determined by the investigators, in safety laboratory parameters (haematology, clinical chemistry, and coagulation, described in detail in **Table 3**) at baseline and on day 2, 3, 5, and 11.

The secondary objective of systemic exposure will be evaluated using the following

Secondary outcome:

- TCP-25 plasma concentrations as measured at Day 1 (before blister formation), day 2 (before administration of the intervention and 0.5 and 1 hours after administration of the

For peer review only - http://bmjopen.bmj.com/site/about/guidelines.xhtml

intervention), day 3 (before administration of the intervention and 1 hour after administration of the intervention) and Day 5 (before administration of the intervention).

Data collection

All outcomes will be assessed by the investigator, who is a physician trained in good clinical practice and qualified to assess these outcomes.

Local tolerability: The investigator will evaluate tolerability by noting abnormal reactions by direct assessment of:

- Skin and wound erythema (abnormal reactions noted)
- Skin and wound oedema and swelling (abnormal reactions noted)
- Wound necrosis, crusting, and haemorrhage (abnormal reactions noted)
- Wound purulent discharge as sign of excessive bacterial colonization and/or infection

The result (yes/no) for each parameter and time point will be documented in the eCRF. Local tolerability parameters, which are judged by the investigator to be aggravated compared to what is expected for blister-induced epidermal wounds, will be reported as AEs.

ECG: Single 12-lead ECG will be recorded in supine position after 10 minutes of rest using an ECG machine. Heart rate (HR), PR, QRS, and QT intervals, and corrected QT interval by Fredericia (QTcF) will be recorded. Safety ECGs will be reviewed and interpreted on-site by the investigator. Any abnormalities will be specified and documented as not clinically significant or clinically significant. Abnormal post-dose findings assessed by the investigator as clinically significant will be reported as AEs.

Vital signs: Will be assessed in the sitting position after 10 minutes of rest. Any vital signs outside the normal ranges will be judged as not clinically significant or clinically significant. The assessment will be recorded in the eCRF. Vital signs judged by the investigator as "abnormal, clinically significant" after application of the intervention will be reported as AEs.

Physical examination: A physical examination will include assessments of general condition, lymph nodes, throat, heart, lungs, and abdomen. Any abnormalities will be specified and

For peer review only - http://bmjopen.bmj.com/site/about/guidelines.xhtml

documented as clinically significant or not clinically significant. Abnormal post-dose findings assessed by the Investigator as clinically significant will be reported as AEs.

Safety lab measurements: Blood will be collected with a peripheral venous catheter using the standard clinical operating procedure and measured by the clinical chemistry department at Skåne University Hospital, Lund, using standard clinical assays. The safety laboratory parameters measured are detailed in **table 1**.

Adverse events: The grading of the intensity of AEs will follow the Common Terminology Criteria for Adverse Events v5.0 [25]. All AEs, including those AEs defined above, shall be recorded. AE will be assessed for causality and severity by the investigator, and any suspected unexpected severe adverse reactions will be reported to the local competent authority and ethics committee according to local and European regulations.

TCP-25 plasma concentrations: Samples for determination of plasma concentrations of TCP-25, will be analysed by Q&Q Laboratories AB, Göteborg, Sweden, by means of a validated LC-MS/MS method. The LLOQ is 30 nmol/L.

These interventions and measurements will be done according to the schedule presented in **Figure 1.**

Sample size and recruitment

No formal sample size calculation has been performed for this study because the study objectives involve safety with few events expected. The proposed sample size is considered sufficient to provide adequate information for the study objectives. Approximately 50 subjects will be screened to achieve 24 randomised and evaluable subjects. The subjects will be healthy volunteers recruited from the Clinical Trial Unit's database of healthy volunteers, using the Unit's regular routines for recruiting. Briefly, potential candidates are contacted by email or phone and provided information about the study. Candidates who sign up for the study are then contacted with further information, followed by screening as described above. The text approved by the independent ethics committee (**supplement 5**, in Swedish) will be used to create all the materials for recruitment.

For peer review only - http://bmjopen.bmj.com/site/about/guidelines.xhtml

Allocation

Each subject will have four suction blister wounds, two on each thigh. Wounds will be labelled R1 (right thigh, distal), R2 (right thigh, proximal), L1 (left thigh, distal) and L2 (left thigh, proximal).

A computer-generated randomisation list will be created using SAS Proc Plan, SAS Version 9.4 by a designated responsible person at Clinical Trial Consultants AB. The randomisation list will contain subject number, wound position, thigh (left/right) and treatment and will be kept by the randomizer in a sealed envelope until database lock. On Day 1 the subjects in each dose group (n=8) will be randomised with regards to the treatment allocation of each wound. Two wounds per patient will receive TCP-25, and the other two will receive placebo. The proximal wound (R1 or L1) of one thigh and the distal wound of the other thigh (L2 or R2) will receive the same treatment, according to the assigned treatment allocation.

An unblinded research nurse in a nearby medical room will read the randomisation list, which indicates the assigned allocation of TCP-25 and control gel to the various wounds for each patient. The research nurse will then fill two syringes per patient. One syringe will contain TCP-25 gel and the other will contain placebo gel. Both syringes will be labelled with the randomisation number of the patient. Additionally they will be labelled with either “R1+L2” or “R2+L1”, corresponding to the two wounds (right proximal + left distal or right distal + left proximal) that are to receive the contents of that syringe according to the assigned treatment allocation. These pre-filled and labelled syringes will be provided to the blinded research staff who will then deposit their contents onto the correct combination of wounds indicated on the syringe label. In this way, the allocation will be communicated to the research staff while ensuring that they remain blinded to the treatment given to each wound.

If needed for emergency unblinding, sealed individual treatment code envelopes will be kept at the clinic and at a contract research organisation (Clinical Trial Consultants AB’s Pharmacovigilance department) in locked and restricted areas.

Blinding

This is a double-blind study and the allocation of treatments will not be disclosed until clean file has been declared and the database has been locked. The TCP-25 and placebo gels are identical in appearance.

For peer review only - http://bmjopen.bmj.com/site/about/guidelines.xhtml

Unblinding will only happen in the following situations:

1. The treatment code may only be broken by the Principal Investigator or delegate in case of emergency when knowledge of the treatment received is necessary for the proper medical management of the subject.
2. The randomization code may be broken by the iSRC during the assessment process (partial unblinding) to enable their decision on continued dosing of further cohorts. The medical staff and subjects will remain blinded to the treatment allocation.

Data management

The data management routines include procedures for handling of the eCRF, database set-up and management, data entry and verification, data validation, QC of the database, and documentation of the performed activities including information of discrepancies in the process. Data validation/data cleaning procedures are designed to assure validity and accuracy of clinical data and consist of computerised online edit checks such as range checks and batch checks on data exports. All study-specific and standard data validation programming will be tested in a separate testing environment prior to use on production data. Detailed information on data management is described in a study-specific Data Management Plan (DMP).

Clinical data will be entered into an eCRF provided by Viedoc Technologies AB. Clinical data will be entered directly from the source documents or at bedside by authorised site personnel designated by the Investigator. All entries of data, corrections, and alterations in the eCRF are to be made by the Investigator or designee, who is to be a qualified physician trained in good clinical practice.

Because data entry is single, all data will undergo source data verification by monitors according to a risk-based monitoring plan. Monitors will review the eCRFs for completeness and consistency and compare data with the respective source documents. If corrections are needed, queries will be raised within the eCRF, either as a result of built-in edit checks or manually and corrected by the site staff. When all data have been entered and discrepancies solved, clean file will be declared, the database will be locked and the code will be broken. The data analysis will begin in the April 2023.

For peer review only - http://bmjopen.bmj.com/site/about/guidelines.xhtml

Statistical analysis plan

Continuous data will be presented in terms of evaluable and missing observations, arithmetic mean, standard deviation (SD), median, minimum, and maximum value. Categorical data will be presented as counts and percentages. When applicable, summary data will be presented by treatment, and by assessment time. Individual subject data will be listed by subject number, treatment, and, where applicable, by assessment time. All descriptive summaries and statistical analyses will be performed using SAS Version 9.4 or later (SAS Institute, Inc.). Baseline will be defined as the last non-missing observation prior to the first administration of the intervention.

Outliers will be included in summary tables and listings and will not be handled separately in any analyses. Generally, no imputation of missing data will be performed. However, clinical safety laboratory parameters that are outside the detection limit, will be replaced with the detection limit when calculating statistics. Also, when calculating statistics for pharmacokinetics plasma concentrations, concentrations under LLOQ will be replaced with LLOQ if more than 50% of the values for a given time point is above LLOQ. Otherwise, no statistics will be calculated for that time point.

Data Monitoring

Internal safety review committee (iSRC)

Before initiating a new dose group, all subjects in the previous dose group must have been treated and safety, tolerability, and pharmacokinetics data (at least data up to 5 days for pharmacokinetics and 8 days for safety) for all treated subjects must have been evaluated by the iSRC. In case of non-replaced dropouts, available data for the dropouts will be included in the iSRC evaluation.

If TCP-25 is considered safe and tolerable, a written recommendation on the next dose level will be provided to the Sponsor.

The voting members of the iSRC will consist of the principal investigator or delegate and the chief physician at the Clinical Trial Unit or delegate. In addition, the study clinical research manager, the study pharmacokineticist, and additional Sponsor representatives may be invited as appropriate. Further internal or external experts may be invited and consulted by the iSRC as appropriate.

For peer review only - http://bmjopen.bmj.com/site/about/guidelines.xhtml

The recommendation to the Sponsor may be study progression, to continue with a higher or lower dose than the intended dose, to repeat the same dose level, to continue with an intermediate dose level, or to stop dosing. The recommendation of the iSRC on the next dose level will be made in consensus between the iSRC members and documented as appropriate.

In case there is disagreement between the 2 voting members, the most conservative approach will be taken.

Auditing

The sponsor has employed a contract research organisation (Clinical Trial Consultants AB) as an independent monitor. Monitoring will be done according to a risk-based monitoring plan and the responsible monitor will periodically visit the study site at times agreed upon by the investigator and the monitor. At each monitoring visit, the role of the Monitor includes, but is not limited to:

- confirm that facilities and resources remain acceptable.
- confirm that the investigational team is adhering to the study protocol, applicable standard operating procedures, guidelines, manuals, and regulatory requirements.
- verify that data are being recorded in the eCRFs in an accurate and timely manner and that accountability checks are being performed.
- verify that data in the eCRF are consistent with the clinical records in accordance with the monitoring plan.
- verify that the correct informed consent procedure has been adhered to for participating subjects.
- verify that AEs are recorded and reported in a timely manner and according to the CSP.
- raise and escalate any serious quality issues, serious GCP breach and any data privacy breach to the Sponsor.

Centralised monitoring will also be performed continuously by study team members at Clinical Trial Consultants AB in accordance with the monitoring plan. When the study has been completed and all queries have been resolved and the database has been locked, the Monitor will perform a close-out visit.

For peer review only - http://bmjopen.bmj.com/site/about/guidelines.xhtml

Authorised representatives of the Sponsor, a competent authority, or an IEC may also perform audits or inspections at the research clinic, including source data verification.

Ethics and dissemination:

Ethical conduct of the study

The study will be performed in accordance with ethical principles that have their origin in the Declaration of Helsinki and are consistent with ICH/GCPE6 (R2), EU Clinical Trials Directive, and applicable local regulatory requirements. The principal investigator is responsible for submitting the study protocol, the subject information and informed consent form, any other written information to be provided to the subjects, and any advertisements used for subject recruitment to the Swedish Ethical Review Authority for approval. The study has been approved by the Swedish Ethical Review Authority (registration number 2022-00527-01)

Before performing any study-related procedures the informed consent form shall be signed and personally dated by the subject and by the investigator. The subjects will be covered under the sponsors liability insurance policy. The participating subjects are also protected in accordance with national regulations, as applicable.

Subject data protection

The informed consent form includes information, that data will be recorded, collected, and processed and may be transferred to other European Economic Area (EEA) or non-EEA countries. In accordance with the General Data Protection Regulation (GDPR) (EU) 2016/679, the data will not identify any persons taking part in the study.

Clinical study report

A summarising report shall be submitted to the Swedish competent authority and IEC within 12 months after completion of the study. A clinical study report, in compliance with ICH-E3, describing the conduct of the study, any statistical analyses performed, and the results obtained, will be prepared. The report will be reviewed and approved by, as a minimum, the principal investigator, the statistician, and the sponsor. The study results will be reported in the EudraCT database per applicable regulations within 12 months after completion of the study. All data obtained from any exploratory analyses will be reported separately.

For peer review only - http://bmjopen.bmj.com/site/about/guidelines.xhtml

Publication and dissemination

Publication of the study results and access to the study data is at the discretion of the Sponsor. Authorship for any publications will be according to the Vancouver criteria. Subjects will be asked to provide a release for use of their photographs in publications if applicable. The subject has a right to refuse the photo release without jeopardising their eligibility to participate in the study.

Patient and public involvement

Patients or the public were neither involved in the design nor will they be involved in this clinical trial's conduct, reporting or dissemination plans.

Protocol version: Protocol version 2.0; 25 January 2022. Protocol amendments will be promptly communicated to all relevant parties, trial registries, and to the ethics committee and competent authority as required by law.

Acknowledgments: We acknowledge the support of the staff at the Clinical Trial Unit at Skane University Hospital Lund and Clinical Trial Consultants AB for input on the Clinical Study Protocol. Xinnate AB provided the project management resources and additional expertise for the regulatory development of TCP-25 gel and the completion of the Clinical Study Protocol. We thank Anne Nielsen and other personnel at the Department of Dermatology Lund and the Clinical Trial Unit at Skane University Hospital Lund for support with the clinical parts of the study. We used the SPIRIT checklist when writing this report (12).

Authors' contributions: SL prepared the first draft of the manuscript, contributed to the development of the clinical study protocol, and revised and approved the final manuscript. KW prepared the first draft of the manuscript, contributed to the development of the clinical study protocol, and revised and approved the final manuscript. JF contributed to the development of the clinical study protocol and revised and approved the final manuscript. SE contributed to the development of the clinical study protocol and revised and approved the final manuscript. AS was responsible for the conception of the study, contributed to the development of the clinical study protocol, and revised and approved the final manuscript. KS

For peer review only

For peer review only - http://bmjopen.bmj.com/site/about/guidelines.xhtml

was responsible for the conception of the study, contributed to the development of the clinical study protocol, and revised and approved the final manuscript.

Roles and responsibilities: A contract research organization (Clinical Trial Consultants AB) will execute the study. the principal investigator and iSRC will carry out analysis, and interpretation of the data. The study sponsor, Xinnate AB, has ultimate responsibility for design of this study, its execution, analyses, interpretation of the data, and decision to submit results.

Funding: The Safety study described here was funded by Xinnate AB. Exploratory analyses on data and materials originating from this study will be integrated with other research projects funded by research grants from the Swedish Research Council (project 2020-02016), Edvard Welanders Stiftelse and Finsenstiftelsen (Hudfonden), the Österlund Foundation, and the Swedish Government Funds for Clinical Research (ALF).

Competing interests: AS is a co-founder and shareholder of Xinnate AB. The other authors report no competing interests.

For peer review only

For peer review only - http://bmjopen.bmj.com/site/about/guidelines.xhtml

Figure legends and tables

Figure 1. Overview of the study design. Wound photography, wound swab/filter, and dressing collection are samples to be collected and biobanked for future exploratory studies. IMP = investigational medicinal product (TCP-25); AE = adverse events; ECG = electrocardiogram

Table 1. Trial registration data

Data category	Information
**Primary registry and trial identifying number**	ClinicalTrials.gov NCT05378997
**Date of registration in primary registry**	18th of May 2022, first submitted 25th of April 2022
**Secondary identifying numbers**	EudraCT 2021-004728-14 (not public)
**Source(s) of monetary or material support**	Xinnate AB,
**Primary sponsor**	Xinnate AB
**Secondary sponsors**	None
**Contact for public queries**	Karim Saleh, MD, PhD<br>Email: karim.saleh@med.lu.se
**Contact for scientific queries**	Karim Saleh, MD, PhD (principal investigator)<br>Email: karim.saleh@med.lu.se
**Public title**	Safety, Tolerability, and Pharmacokinetics of Ascending Topical Doses of TCP-25 Applied to Epidermal Suction Blister Wounds
**Scientific title**	A Phase 1, Randomized, Double-Blind, Placebo-Controlled Study in Healthy Male and Female Volunteers to Investigate the Safety, Tolerability, and Pharmacokinetics of Ascending Topical Doses of TCP-25 Applied to Epidermal Suction Blister Wounds
**Countries of recruitment**	Sweden
**Health condition(s) or problem(s) studied**	Acute epidermal wounds
**Intervention(s)**	Active comparator: TCP-25 gel (0.86, 2.9, or 8.6 mg/mL)
	Placebo comparator: placebo gel (equivalent gel with no TCP-25)

Data category	Information
**Key inclusion and exclusion criteria**	Ages eligible for study: 18-60 years<br>Sexes eligible for study: both<br>Accepts healthy volunteers: yes
	Inclusion criteria: See table 2
	Exclusion criteria: See table 2
**Study type**	Interventional
	Allocation: randomized<br>Model: Sequential assignment (ascending doses)<br>Masking: Quadruple (Participant, Care Provider, Investigator, Outcomes Assessor)
	Primary purpose: Treatment
	Phase I
**Date of first enrolment**	April 7, 2022
**Target sample size**	24
**Recruitment status**	Recruiting
**Primary outcome(s)**	Adverse Events [Time Frame: 15 days]<br>Incidence of abnormal local reactions (Local tolerability) [Time Frame: Day 1, 2, 3, 5, 8, 11]<br>Number of patients with clinically significant changes from baseline in electrocardiogram (ECG), vital signs, and physical examinations [Time Frame: During screening (baseline) and on day 11]<br>Number of patients with clinically significant changes from baseline in safety laboratory parameters [Time Frame: During screening (baseline) and on day 2, 3, 5, 11]
**Key secondary outcomes**	Plasma concentration of TCP-25 [Time Frame: Day 1 (measured before blister formation), Day 2 (measured before administration of the intervention and 0.5 and 1 hours after administration of the intervention), Day 3 (measured before administration of the intervention 1 hour after administration of the intervention), and Day 5 (measured before administration of intervention)
**Ethics review**	Approved 2022 March 10, Eti kprövningsmyndigheten registration number 2022-00527-01

Table 2. Summary of inclusion and exclusion criteria

Inclusion criteria	Exclusion criteria
1. Willing and able to give written informed consent for participation in the study.<br>2. Healthy male or female subject 18-60 years (inclusive) of age at the time of signing the informed consent.<br>3. Body Mass Index (BMI) ≥ 18.0 and ≤ 30.0 kg/m2.<br>4. Healthy and intact skin where the blister suction wounds will be induced.<br>5. Women of childbearing potential must have a documented negative serum pregnancy test done at the screening visit, within 4 weeks prior to suction blister formation and the start of study treatment.<br>Male subjects must be willing to use condom or be vasectomised or practice sexual abstinence to prevent pregnancy.<br>6. Clinically relevant medical history, physical findings, vital signs, ECG and laboratory values at the time of screening.	1. History of any clinically significant disease or disorder.<br>2. Any disease that may interfere with wound healing.<br>3. Active skin disease and/or tattoos in the areas where suction blister wounds will be induced.<br>4. Any planned major surgery within the duration of the study.<br>5. Any vital sign values outside the normal range.<br>6. Any clinically significant abnormalities in the resting ECG at the time of screening.<br>7. Current smokers or users of nicotine products.<br>8. Female subjects who are pregnant or lactating or planning a pregnancy.<br>9. Systemic immunosuppressive treatment<br>10. Subjects who are currently receiving or have received antibiotics or systemic corticosteroids within 2 weeks prior to screening.<br>11. Regular use of anticoagulants or non-steroidal anti-inflammatory drugs (NSAIDs) within 2 weeks prior to the (first) administration of the intervention.<br>12. History of or ongoing severe allergy/hypersensitivity to drugs with a similar chemical structure or class to TCP-25 or to any excipients<br>13. Planned treatment or treatment with another investigational drug within 3 months prior to Day -1<br>14. History of alcohol abuse or excessive intake of alcohol

For peer review only - http://bmjopen.bmj.com/site/about/guidelines.xhtml

	15. Presence or history of drug abuse<br>16. Plasma donation within one month of screening or blood donation (or corresponding blood loss) during the three months prior to screening<br>17. Involvement in the planning and/or conduct of the study<br>18. Unlikely to comply with study procedures, restrictions and requirements

Table 3. Safety laboratory parameters

Category	Parameter
**Clinical chemistry**	Alanine aminotransferase (ALT)
	Aspartate aminotransferase (AST)
	Creatinine
	C-reactive protein (CRP)
	Glucose
	HbA1C
**Haematology**	Haematocrit
	Haemoglobin (Hb)
	Erythrocytes
	Mean corpuscular volume (MCV)
	Mean corpuscular haemoglobin (MCH)
	Mean corpuscular haemoglobin concentration (MCHC)
	Thrombocytes
	Leukocytes
	Eosinophils
	Neutrophils
	Basophils
	Lymphocytes
	Monocytes
**Coagulation**	Activated partial thromboplastin time (APTT)<br>Prothrombin complex/International Normalised Ratio (PK/INR)
**Pregnancy test (WOCBP only)**	Serum pregnancy test at screening
	Urine pregnancy test (Visit 2)

For peer review only

For peer review only - http://bmjopen.bmj.com/site/about/guidelines.xhtml

References

1. Eming SA, Krieg T, Davidson JM. Inflammation in Wound Repair: Molecular and Cellular Mechanisms. Journal of Investigative Dermatology. 2007;127(3):514-25.
2. Sen CK, Gordillo GM, Roy S, Kirsner R, Lambert L, Hunt TK, et al. Human skin wounds: a major and snowballing threat to public health and the economy. Wound Repair Regen. 2009;17(6):763-71.
3. Nathan C, Cars O. Antibiotic Resistance — Problems, Progress, and Prospects. New England Journal of Medicine. 2014;371(19):1761-3.
4. Filius PMG, Gyssens IC. Impact of Increasing Antimicrobial Resistance on Wound Management. American Journal of Clinical Dermatology. 2002;3(1):1-7.
5. Michael CA, Dominey-Howes D, Labbate M. The antimicrobial resistance crisis: causes, consequences, and management. Front Public Health. 2014;2:145.
6. Puthia M, Butrym M, Petrlova J, Strömdahl AC, Andersson M, Kjellström S, et al. A dual-action peptide-containing hydrogel targets wound infection and inflammation. Sci Transl Med. 2020;12(524).
7. Strömdahl AC, Ignatowicz L, Petruk G, Butrym M, Wasserstrom S, Schmidtchen A, et al. Peptide-coated polyurethane material reduces wound infection and inflammation. Acta Biomater. 2021;128:314-31.
8. Kottner J, Hillmann K, Fimmel S, Seité S, Blume-Peytavi U. Characterisation of epidermal regeneration in vivo: a 60-day follow-up study. J Wound Care. 2013;22(8):395-400.
9. Ahlström MG, Gjerdrum LMR, Larsen HF, Fuchs C, Sørensen AL, Forman JL, et al. Suction blister lesions and epithelialization monitored by optical coherence tomography. Skin Res Technol. 2018;24(1):65-72.
10. Daeschlein G, Alborova J, Patzelt A, Kramer A, Lademann J. Kinetics of physiological skin flora in a suction blister wound model on healthy subjects after treatment with water-filtered infrared-A radiation. Skin Pharmacol Physiol. 2012;25(2):73-7.
11. Burian EA, Sabah L, Kirketerp-Møller K, Gundersen G, Ågren MS. Effect of Stabilized Hypochlorous Acid on Re-epithelialization and Bacterial Bioburden in Acute Wounds: A Randomized Controlled Trial in Healthy Volunteers. Acta Derm Venereol. 2022;102:adv00727.
12. Chan A-W, Tetzlaff JM, Gøtzsche PC, Altman DG, Mann H, Berlin JA, et al. SPIRIT 2013 explanation and elaboration: guidance for protocols of clinical trials. BMJ : British Medical Journal. 2013;346:e7586.
13. Larsen HF, Ahlström MG, Gjerdrum LMR, Mogensen M, Ghathian K, Calum H, et al. Noninvasive measurement of reepithelialization and microvascularity of suction-blister wounds with benchmarking to histology. Wound Repair Regen. 2017;25(6):984-93.

For peer review only - http://bmjopen.bmj.com/site/about/guidelines.xhtml

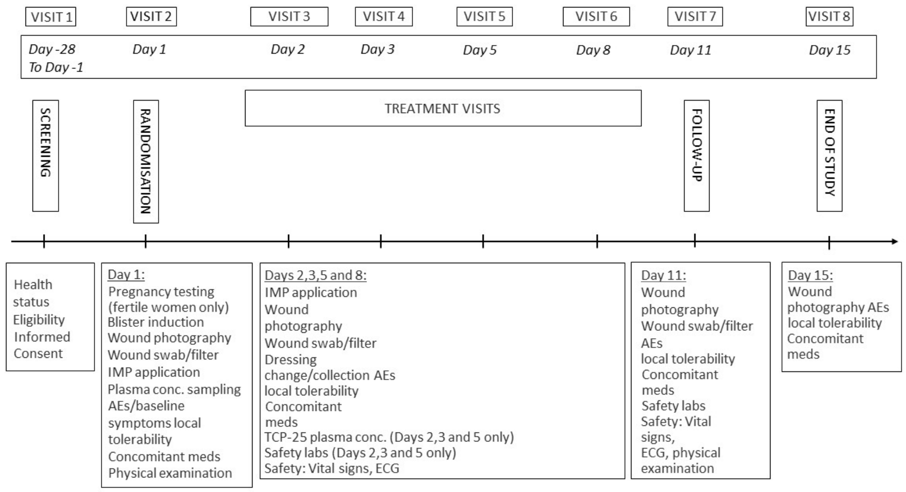

Figure 1. Overview of the study design. Wound photography, wound swab/filter, and dressing collection are samples to be collected and biobanked for future exploratory studies. IMP = investigational medicinal product (TCP-25); AE = adverse events; ECG = electrocardiogram

338x190mm (96 x 96 DPI)

Supplement 1, Full inclusion and exclusion criteria

Inclusion criteria	Exclusion criteria
1. Willing and able to give written informed consent for participation in the study.	1. History of any clinically significant disease or disorder which, in the opinion of the Investigator, may either put the subject at risk because of participation in the study, or influence the results or the subject's ability to participate in the study.
2. Healthy male or female subject 18-60 years (inclusive) of age at the time of signing the informed consent.	2. Disease that may interfere with wound healing, e.g., diabetes type I/II, arterial-, renal-, liver, or cardiac insufficiency, chronic obstructive lung disease, cancer, autoimmune disease, edema at the study site, severe obesity, or previous known wound healing problems, as judged by the investigator.
3. Body Mass Index (BMI) $\geq$ 18.0 and $\leq$ 30.0 kg/m2.	3. Active skin disease, e.g., dermatitis, psoriasis and wounds, and/or tattoos in the areas where suction blister wounds will be induced, as judged by the investigator.
4. Healthy and intact skin where the blister suction wounds will be induced.	4. Any planned major surgery within the duration of the study.
5. Women of childbearing potential (WOCBP) must have a documented negative serum pregnancy test done at the screening visit, within 4 weeks prior to suction blister formation and the start of study treatment.<br>WOCBP must practice abstinence (only allowed when this is the preferred and usual lifestyle of the subject) or must agree to use a highly effective method of contraception with a failure rate of < 1% to prevent pregnancy<br>Male subjects must be willing to use condom or be vasectomised or practice sexual abstinence to prevent pregnancy and drug exposure of a partner and refrain from donating sperm from the date of last dosing until 3 months after the last dosing with the IMP. Their female partner of child-bearing potential must use contraceptive methods with a failure rate of < 1% to prevent pregnancy (see above).	5. After 10 minutes supine rest at the time of screening, any vital signs values outside the following ranges:<br>• Systolic blood pressure 160 mmHg, or<br>• Diastolic blood pressure 100 mmHg, or<br>• Pulse 90 beats per minute (bpm)
6. Clinically relevant medical history, physical findings, vital signs, ECG and laboratory values at the time of screening, as judged by the Investigator.	6. Any clinically significant abnormalities in the resting ECG at the time of screening, as judged by the Investigator.
	7. Current smokers or users of nicotine products. Irregular use of nicotine (e.g., smoking, snuffing, chewing tobacco) less than three times per week is allowed before screening visit.
	8. Female subjects who are pregnant or lactating or planning a pregnancy.
	9. Systemic immunosuppressive treatment
	10. Subjects who are currently receiving or have received the following treatments within 2 weeks prior to screening are excluded from the

For peer review only

	study: - systemic corticosteroids or immunosuppressant agents; or - antibiotics via any route
	11. Regular use of anticoagulants (i.e., heparin, warfarin, coumarins, other anticoagulants per Investigator's judgement) or non-steroidal anti-inflammatory drugs (NSAIDs) within 2 weeks prior to the (first) administration of IMP, at the discretion of the Investigator.
	12. History of severe allergy/hypersensitivity or ongoing allergy/hypersensitivity, as judged by the Investigator, or history of hypersensitivity to drugs with a similar chemical structure or class to TCP-25 or to any excipients of the hydrogel.
	13. Planned treatment or treatment with another investigational drug within 3 months prior to Day -1.
	14. History of alcohol abuse or excessive intake of alcohol, as judged by the Investigator.
	15. Presence or history of drug abuse, as judged by the Investigator
	16. Plasma donation within one month of screening or blood donation (or corresponding blood loss) during the three months prior to screening.
	17. Involvement in the planning and/or conduct of the study.
	18. Investigator considers the subject unlikely to comply with study procedures, restrictions and requirements.

For peer review only

Supplement 2, Restrictions during the study

- Contraception Requirements: The male volunteers are expected to use condom to prevent pregnancy and drug exposure of a partners and refrain from donating sperm from the date of dosing until 4 weeks after the last dosing of the IMP.
  Fertile female study subjects are expected to practice abstinence or use contraceptive methods with a failure rate of < 1% to prevent pregnancy during the same period, i.e., combined [oestrogen and progestogen containing] hormonal contraception associated with inhibition of ovulation [oral, intravaginal, transdermal], progestogen-only hormonal contraception associated with inhibition of ovulation [oral, injectable, implantable], IUD or intrauterine IUS) from at least 4 weeks prior to dose to 4 weeks after last dose.
- Caffeine: Consumption of caffeine equivalent to up to 5 cups of coffee per day will be allowed from screening to the end-of-study visit.
- Other xanthine or taurine containing products/beverages than tea or coffee: Energy drinks (e.g., Red Bull, Battery, Flying Horse, La Bomba) are not allowed from screening to the end-of-study visit.
- Nicotine: Smoking (>3 cigarettes per day) or use of nicotine-containing products is not allowed from screening to the end-of-study visit.
- Exercise: From Day 1 to Day 11, the subjects must abstain from strenuous exercise that would cause excessive sweating compromising the dressing of the wounds.
- Blood donation: The subjects must not donate blood or plasma during the study until 3 months after the final medical examination at the end-of-study visit.
- Participation in other clinical studies: Study subjects are not allowed to participate in any other interventional clinical study during the study period.

Supplement 3, Prior and concomitant therapy

Prohibited medication

From 2 weeks prior to wounding and throughout the study until Visit 9 (unless indicated for treatment of any AEs):

- Systemic corticosteroids or immunosuppressant agents; or
- Antibiotics via any route
- Anticoagulants (e.g., heparin, warfarin, coumarins, other anticoagulants per Investigator's judgement)
- Regular use of NSAIDs (i.e., ibuprofen, diclofenac, acetylsalicylic acid and others)

Allowed medication

- Paracetamol 1 g up to 4 times daily is allowed for treatment of post-procedural pain.
- Any medications at a stable dose to treat medical history events will be allowed throughout the study.
- Medications considered necessary for the subject´s safety and wellbeing may be given at the discretion of the Investigator during the study. Following consultation with the Sponsor, the Investigator will determine whether or not the subject should continue in the study.

For peer review only

Supplement 4, General withdrawal criteria

Subjects are free to discontinue their participation in the study at any time and for whatever reason without affecting their right to an appropriate follow-up investigation or their future care. If possible, the reason for withdrawal of consent should be documented.

Subjects may be discontinued from the study at any time at the discretion of the Investigator.

Reasons for discontinuation include:

- Subject decision
- Severe non-compliance to study protocol procedures, as judged by the Investigator and/or Sponsor
- Subject is lost to follow-up.
- Significant AEs posing a risk for the subject, as judged by the Investigator and/or Sponsor
- Withdrawal of informed consent to the use of biological samples
- Pregnancy
- Death
- Meeting of an exclusion criterion during the study, which, in the opinion of the Investigator, may pose a risk for the subject
- Use of prohibited medication

For peer review only

Supplement 5 – text for recruitment and advertisement approved by the ethics committee

Long version:

En fas 1, randomiserad, dubbelblind och kontrollerad studie på friska män och kvinnor för att undersöka säkerhet, tolerans och exponering efter applikation av stigande doser av TCP-25, en gel som appliceras utvärtes på ytliga sår i huden

Syftet med denna Fas 1 studie är att undersöka läkemedlets säkerhet och tolerans

Kriterier för deltagande:

• Du ska vara frisk

• Du får ej ha högt blodtryck eller diabetes

• Du får inte ha födelsemärken, tatueringar eller hudförändringar på låren.

• Du får inte vara överviktig eller underviktig (BMI ska ligga mellan 18–30 kg/m2)

• Du får ej röka/snusa regelbundet

• Du får ej ha deltagit i annan läkemedelsstudie senaste 3 månaderna

• Du får inte ha lämnat plasma 1 månad eller blod 3 månader innan den inledande hälsokontrollen för studien.

• Om du är i fertil ålder och inte steril måste du 4 veckor efter dosering av studieläkemedel använda dubbelbarriär vid heterosexuella samlag, dvs kondom/godkända preventivmedel. Är du kvinna gäller detta även fyra veckor innan studiestart.

I studien sker totalt 8 besök på Klinisk Prövningsenhet, Skånes Universitetssjukhus i Lund, under en period av 15 dagar. Du kommer få 2 små blåsor, 2 på vardera lår (totalt 4 blåsor).

Blåsorna skapas med hjälp av en pump som skapar undertryck och liknas de blåsor som uppstår vid skoskav både till utseende och djup. Blåstaket och vätskan avlägsnas och ett sår med en diameter på 1 cm uppstår, studieläkemedel/placebo ges sedan lokalt på såren.

Du får ersättning för ditt deltagande. Vill du delta eller ha mer information? Maila provningsenheten.forskningsstudie@skane.se eller besök https://sodrasjukvardsregionen.se/kliniskastudier/delta-i-klinisk-studie/ Studien är godkänd av Etikprövningsmyndigheten och Läkemedelsverket. Din medverkan är frivillig och du kan när som helst avbryta ditt deltagande. Den information du lämnar kommer att behandlas i enlighet med Dataskyddsförordningen (GDPR).

For peer review only

Short version:

En fas 1, randomiserad, dubbelblind och kontrollerad studie på friska män och kvinnor för att undersöka säkerhet, tolerans och exponering efter applikation av stigande doser av TCP-25, en gel som appliceras utvärtes på ytliga sår i huden

Syftet med studien är att undersöka läkemedlets säkerhet och tolerans samt hur läkemedlet tas upp, fördelas och utsöndras i kroppen.

Du får ersättning för ditt deltagande samt reseersättning.

För att kunna delta behöver du vara frisk och ej ha några allvarliga sjukdomar. Vill du delta eller ha mer information? Maila provningsenheten.forskningsstudie@skane.se eller besök https://sodrasjukvardsregionen.se/kliniskastudier/delta-i-klinisk-studie/

Studien är godkänd av Etikprövningsmyndigheten och Läkemedelsverket. Din medverkan är frivillig och du kan när som helst avbryta ditt deltagande. Den information du lämnar kommer att behandlas i enlighet med Dataskyddsförordningen (GDPR).

For peer review only

Reporting checklist for protocol of a clinical trial.

Based on the SPIRIT guidelines.

Instructions to authors

Complete this checklist by entering the page numbers from your manuscript where readers will find each of the items listed below.

Your article may not currently address all the items on the checklist. Please modify your text to include the missing information. If you are certain that an item does not apply, please write "n/a" and provide a short explanation.

Upload your completed checklist as an extra file when you submit to a journal.

In your methods section, say that you used the SPIRITreporting guidelines, and cite them as:

Chan A-W, Tetzlaff JM, Gøtzsche PC, Altman DG, Mann H, Berlin J, Dickersin K, Hróbjartsson A, Schulz KF, Parulekar WR, Krleža-Jerić K, Laupacis A, Moher D. SPIRIT 2013 Explanation and Elaboration: Guidance for protocols of clinical trials. BMJ. 2013;346:e7586

		Reporting Item	Page Number
**Administrative information**			
Title	#1	Descriptive title identifying the study design, population, interventions, and, if applicable, trial acronym	Page 1
Trial registration	#2a	Trial identifier and registry name. If not yet	Page 2

For peer review only

		registered, name of intended registry	
Trial registration: data set	#2b	All items from the World Health Organization Trial Registration Data Set	Table 1
Protocol version	#3	Date and version identifier	Page 13
Funding	#4	Sources and types of financial, material, and other support	Page 13
Roles and responsibilities: contributorship	#5a	Names, affiliations, and roles of protocol contributors	Page 14
Roles and responsibilities: sponsor contact information	#5b	Name and contact information for the trial sponsor	Page 13
Roles and responsibilities: sponsor and funder	#5c	Role of study sponsor and funders, if any, in study design; collection, management, analysis, and interpretation of data; writing of the report; and the decision to submit the report for publication, including whether they will have ultimate authority over any of these activities	Page 13
Roles and responsibilities: committees	#5d	Composition, roles, and responsibilities of the coordinating centre, steering committee, endpoint adjudication committee, data	Page 10-11, Page 13

For peer review only

		management team, and other individuals or groups overseeing the trial, if applicable (see Item 21a for data monitoring committee)	
**Introduction**			
Background and rationale	#6a	Description of research question and justification for undertaking the trial, including summary of relevant studies (published and unpublished) examining benefits and harms for each intervention	Page 3
Background and rationale: choice of comparators	#6b	Explanation for choice of comparators	Page 4
Objectives	#7	Specific objectives or hypotheses	Page 4
Trial design	#8	Description of trial design including type of trial (eg, parallel group, crossover, factorial, single group), allocation ratio, and framework (eg, superiority, equivalence, non-inferiority, exploratory)	Page 4-5
**Methods: Participants, interventions, and outcomes**			
Study setting	#9	Description of study settings (eg, community	Page 4-5

For peer review only

		clinic, academic hospital) and list of countries where data will be collected. Reference to where list of study sites can be obtained	
Eligibility criteria	#10	Inclusion and exclusion criteria for participants. If applicable, eligibility criteria for study centres and individuals who will perform the interventions (eg, surgeons, psychotherapists)	Page 5
Interventions: description	#11a	Interventions for each group with sufficient detail to allow replication, including how and when they will be administered	Page 6
Interventions: modifications	#11b	Criteria for discontinuing or modifying allocated interventions for a given trial participant (eg, drug dose change in response to harms, participant request, or improving / worsening disease)	Page 5
Interventions: adherance	#11c	Strategies to improve adherence to intervention protocols, and any procedures for monitoring adherence (eg, drug tablet return; laboratory tests)	N/A (all study intervention protocols will be carried out by a study nurse, so patient compliance is not a factor).
Interventions: concomitant care	#11d	Relevant concomitant care and interventions that are permitted or prohibited during the trial	Page 6, supplement 2 and 3

For peer review only

Outcomes	#12	Primary, secondary, and other outcomes, including the specific measurement variable (eg, systolic blood pressure), analysis metric (eg, change from baseline, final value, time to event), method of aggregation (eg, median, proportion), and time point for each outcome. Explanation of the clinical relevance of chosen efficacy and harm outcomes is strongly recommended	Page 6-7
Participant timeline	#13	Time schedule of enrolment, interventions (including any run-ins and washouts), assessments, and visits for participants. A schematic diagram is highly recommended (see Figure)	Figure 1
Sample size	#14	Estimated number of participants needed to achieve study objectives and how it was determined, including clinical and statistical assumptions supporting any sample size calculations	Page 8
Recruitment	#15	Strategies for achieving adequate participant enrolment to reach target sample size	Page 8

Methods: Assignment of interventions (for

For peer review only

controlled trials)

Allocation: sequence generation	#16a	Method of generating the allocation sequence (eg, computer-generated random numbers), and list of any factors for stratification. To reduce predictability of a random sequence, details of any planned restriction (eg, blocking) should be provided in a separate document that is unavailable to those who enrol participants or assign interventions	Page 8
Allocation concealment mechanism	#16b	Mechanism of implementing the allocation sequence (eg, central telephone; sequentially numbered, opaque, sealed envelopes), describing any steps to conceal the sequence until interventions are assigned	Page 8
Allocation: implementation	#16c	Who will generate the allocation sequence, who will enrol participants, and who will assign participants to interventions	Page 8
Blinding (masking)	#17a	Who will be blinded after assignment to interventions (eg, trial participants, care providers, outcome assessors, data analysts), and how	Page 9
Blinding (masking): emergency unblinding	#17b	If blinded, circumstances under which unblinding is permissible, and procedure for revealing a participant's allocated intervention	Page 9

For peer review only

		during the trial	
**Methods: Data collection, management, and analysis**			
Data collection plan	#18a	Plans for assessment and collection of outcome, baseline, and other trial data, including any related processes to promote data quality (eg, duplicate measurements, training of assessors) and a description of study instruments (eg, questionnaires, laboratory tests) along with their reliability and validity, if known. Reference to where data collection forms can be found, if not in the protocol	Page 7 - 8
Data collection plan: retention	#18b	Plans to promote participant retention and complete follow-up, including list of any outcome data to be collected for participants who discontinue or deviate from intervention protocols	Page 5, 6
Data management	#19	Plans for data entry, coding, security, and storage, including any related processes to promote data quality (eg, double data entry; range checks for data values). Reference to where details of data management	Page 9

For peer review only

		procedures can be found, if not in the protocol	
Statistics: outcomes	#20a	Statistical methods for analysing primary and secondary outcomes. Reference to where other details of the statistical analysis plan can be found, if not in the protocol	Page 10
Statistics: additional analyses	#20b	Methods for any additional analyses (eg, subgroup and adjusted analyses)	Page 10
Statistics: analysis population and missing data	#20c	Definition of analysis population relating to protocol non-adherence (eg, as randomised analysis), and any statistical methods to handle missing data (eg, multiple imputation)	Page 10
**Methods: Monitoring**			
Data monitoring: formal committee	#21a	Composition of data monitoring committee (DMC); summary of its role and reporting structure; statement of whether it is independent from the sponsor and competing interests; and reference to where further details about its charter can be found, if not in the protocol. Alternatively, an explanation of why a DMC is not needed	Page 10 - 11
Data monitoring: interim analysis	#21b	Description of any interim analyses and stopping guidelines, including who will have access to these interim results and make the	Page 10-11

For peer review only

		final decision to terminate the trial	
Harms	#22	Plans for collecting, assessing, reporting, and managing solicited and spontaneously reported adverse events and other unintended effects of trial interventions or trial conduct	Page 7-8
Auditing	#23	Frequency and procedures for auditing trial conduct, if any, and whether the process will be independent from investigators and the sponsor	Page 11-12
**Ethics and dissemination**			
Research ethics approval	#24	Plans for seeking research ethics committee / institutional review board (REC / IRB) approval	Page 12
Protocol amendments	#25	Plans for communicating important protocol modifications (eg, changes to eligibility criteria, outcomes, analyses) to relevant parties (eg, investigators, REC / IRBs, trial participants, trial registries, journals, regulators)	Page 13
Consent or assent	#26a	Who will obtain informed consent or assent from potential trial participants or authorised surrogates, and how (see Item 32)	Page 5

For peer review only

Consent or assent: ancillary studies	#26b	Additional consent provisions for collection and use of participant data and biological specimens in ancillary studies, if applicable	Page 5
Confidentiality	#27	How personal information about potential and enrolled participants will be collected, shared, and maintained in order to protect confidentiality before, during, and after the trial	Page 12
Declaration of interests	#28	Financial and other competing interests for principal investigators for the overall trial and each study site	Page 14
Data access	#29	Statement of who will have access to the final trial dataset, and disclosure of contractual agreements that limit such access for investigators	Page 13
Ancillary and post trial care	#30	Provisions, if any, for ancillary and post-trial care, and for compensation to those who suffer harm from trial participation	Ancillary and post-trial care: not applicable. Compensation (insurance): Page 12
Dissemination policy: trial results	#31a	Plans for investigators and sponsor to communicate trial results to participants, healthcare professionals, the public, and other relevant groups (eg, via publication, reporting in results databases, or other data sharing	Page 13

For peer review only

		arrangements), including any publication restrictions	
Dissemination policy: authorship	#31b	Authorship eligibility guidelines and any intended use of professional writers	Page 13
Dissemination policy: reproducible research	#31c	Plans, if any, for granting public access to the full protocol, participant-level dataset, and statistical code	N/A (no plans)
**Appendices**			
Informed consent materials	#32	Model consent form and other related documentation given to participants and authorised surrogates	N/A (not included for publication)
Biological specimens	#33	Plans for collection, laboratory evaluation, and storage of biological specimens for genetic or molecular analysis in the current trial and for future use in ancillary studies, if applicable	Page 4

Notes:

The SPIRIT Explanation and Elaboration paper is distributed under the terms of the Creative Commons Attribution License CC-BY-NC. This checklist was completed on 16. May 2022 using https://www.goodreports.org/, a tool made by the EQUATOR Network in collaboration with Penelope.ai

For peer review only
